# Genetic Footprints of Iberian Cattle in America 500 Years after the Arrival of Columbus

**DOI:** 10.1371/journal.pone.0049066

**Published:** 2012-11-14

**Authors:** Amparo M. Martínez, Luis T. Gama, Javier Cañón, Catarina Ginja, Juan V. Delgado, Susana Dunner, Vincenzo Landi, Inmaculada Martín-Burriel, M. Cecilia T. Penedo, Clementina Rodellar, Jose Luis Vega-Pla, Atzel Acosta, Luz A. Álvarez, Esperanza Camacho, Oscar Cortés, Jose R. Marques, Roberto Martínez, Ruben D. Martínez, Lilia Melucci, Guillermo Martínez-Velázquez, Jaime E. Muñoz, Alicia Postiglioni, Jorge Quiroz, Philip Sponenberg, Odalys Uffo, Axel Villalobos, Delsito Zambrano, Pilar Zaragoza

**Affiliations:** 1 Departamento de Genética, Universidad de Córdoba, Córdoba, Spain; 2 L-INIA, Instituto Nacional dos Recursos Biológicos, Fonte Boa, Vale de Santarém, Portugal; 3 CIISA – Faculdade de Medicina Veterinária, Universidade Técnica de Lisboa, Lisboa, Portugal; 4 Departamento de Producción Animal, Facultad de Veterinaria, Universidad Complutense de Madrid, Madrid, Spain; 5 Centre for Environmental Biology, Faculty of Sciences, University of Lisbon & Molecular Biology Group, Instituto Nacional de Recursos Biológicos, INIA, Lisbon, Portugal; 6 Laboratorio de Genética Bioquímica, Facultad de Veterinaria, Universidad de Zaragoza, Zaragoza, Spain; 7 Veterinary Genetics Laboratory, University of California Davis, Davis, California, United States of America; 8 Laboratorio de Investigación Aplicada, Cría Caballar de las Fuerzas Armadas, Córdoba, Spain; 9 Centro Nacional de Sanidad Agropecuaria, San José de las Lajas, La Habana, Cuba; 10 Universidad Nacional de Colombia, Sede Palmira, Valle del Cauca, Colombia; 11 IFAPA, Centro Alameda del Obispo, Córdoba, Spain; 12 EMBRAPA Amazônia Oriental, Belém, Pará, Brazil; 13 Centro Multidisciplinario de Investigaciones Tecnológicas, Dirección General de Investigación Científica y Tecnológica, Universidad Nacional de Asunción, San Lorenzo, Paraguay; 14 Genética Animal, Facultad de Ciencias Agrarias, Universidad Nacional de Lomas de Zamora, Lomas de Zamora, Argentina; 15 Facultad Ciencias Agrarias, Universidad Nacional de Mar del Plata, Balcarce, Argentina; 16 Estación Experimental Agropecuaria Balcarce, Instituto Nacional de Tecnología Agropecuaria, Balcarce, Argentina; 17 Instituto Nacional de Investigaciones Forestales, Agrícolas y Pecuarias, Coyoacán, México; 18 Área Genética, Departamento de Genética y Mejora Animal, Facultad de Veterinaria, Universidad de la República, Montevideo, Uruguay; 19 Virginia-Maryland Regional College of Veterinary Medicine, Virginia Tech, Blacksburg, Virginia, United States of America; 20 Instituto de Investigación Agropecuaria, Estación Experimental El Ejido, Los Santos, Panamá; 21 Universidad Técnica Estatal de Quevedo, Quevedo, Ecuador; Fordham University, United States of America

## Abstract

**Background:**

American Creole cattle presumably descend from animals imported from the Iberian Peninsula during the period of colonization and settlement, through different migration routes, and may have also suffered the influence of cattle directly imported from Africa. The introduction of European cattle, which began in the 18th century, and later of Zebu from India, has threatened the survival of Creole populations, some of which have nearly disappeared or were admixed with exotic breeds. Assessment of the genetic status of Creole cattle is essential for the establishment of conservation programs of these historical resources.

**Methodology/Principal Findings:**

We sampled 27 Creole populations, 39 Iberian, 9 European and 6 Zebu breeds. We used microsatellite markers to assess the origins of Creole cattle, and to investigate the influence of different breeds on their genetic make-up. The major ancestral contributions are from breeds of southern Spain and Portugal, in agreement with the historical ports of departure of ships sailing towards the Western Hemisphere. This Iberian contribution to Creoles may also include some African influence, given the influential role that African cattle have had in the development of Iberian breeds, but the possibility of a direct influence on Creoles of African cattle imported to America can not be discarded. In addition to the Iberian influence, the admixture with other European breeds was minor. The Creoles from tropical areas, especially those from the Caribbean, show clear signs of admixture with Zebu.

**Conclusions/Significance:**

Nearly five centuries since cattle were first brought to the Americas, Creoles still show a strong and predominant signature of their Iberian ancestors. Creole breeds differ widely from each other, both in genetic structure and influences from other breeds. Efforts are needed to avoid their extinction or further genetic erosion, which would compromise centuries of selective adaptation to a wide range of environmental conditions.

## Introduction

*“That many breeds of cattle have originated through variation, independently of descent from distinct species, we may infer from what we see in South America, where the genus Bos was not endemic, and where the cattle which now exist in such vast numbers are the descendants of a few imported from Spain and Portugal.”*

*Charles Darwin, in The Variation of Animals and Plants Under Domestication, 1868*

Columbus’s trip to the Americas was one of the most important events in the history of humanity, as it produced major social and economic changes on both sides of the Atlantic. The Pre-Columbian American civilizations were predominantly agriculturalist but few were livestock keepers. The only domesticated species in the Americas were the dog, turkey, guinea pig and two Andean camelids [1]. One of the major impacts of Columbus’s trip was the exchange of plant and animal genetic resources among continents, which revolutionized the way of life and food habits of populations in both Europe and the Americas [2].

Livestock species were brought from the Iberian Peninsula to the Americas since the late 15th century, starting with the second trip of Columbus, which departed from the Spanish city of Cádiz in 1493. In this trip, which had a re-supply in the Canary Islands, Columbus brought horses, cattle, sheep, goats and pigs to the Americas for the first time [3]. Afterwards, many other conquerors and settlers followed, and cattle brought from the Iberian Peninsula, and possibly directly from Africa at a later stage, spread throughout the Americas, adapting to a wide range of environmental conditions and giving origin to the populations currently known as Creole cattle [4]. After nearly 300 years of expansion of Creole cattle in the American continents, and with the development of more intensive production and breeding systems, several other European breeds were introduced into the Americas in the 19th century [5]. By the end of the 19th century, Indian cattle breeds, of the Zebu or *Bos indicus* type, were also introduced and quickly disseminated throughout the Americas, where they were extensively crossed with local populations, especially in tropical regions [6].

For over three centuries, Creole cattle were used as a source of draught power, food and leather, playing a key role in the settlement of human populations and the development of agriculture throughout the Western Hemisphere [7]. However, the successive introduction of different cattle breeds starting in the 19th century resulted in the progressive replacement of many Creole populations, which have completely disappeared in several regions or were displaced to marginal areas, where they still subsist nowadays [8]. Even though these extant populations present high levels of genetic diversity [9] and result from several centuries of adaptation to local environments, it is not clear how much of the ancestral Iberian founder contributions have been retained, or if the successive waves of other cattle introduced over the years have replaced the original contribution of Iberian stock.

The study of genetic diversity within and across breeds provides insight into population structure and relationships, and is essential for the development of conservation and breeding programs. Microsatellite genetic markers have been extensively used to assess between- and within-breed genetic diversity and inbreeding levels, introgression from other genetic groups, genetic differentiation and population structure [10]–[14]. The phylogeny of cattle has also been investigated with other types of genetic markers, including mtDNA [15], the non-recombining region of the Y chromosome [16] and single nucleotide polymorphisms [17]–[19] The insight on breed development and introgression provided by the different types of genetic markers is complementary, with neutral genetic markers such as microsatellites essentially reflecting the consequences of genetic drift, founder effects and population admixture. This is particularly important in the case of Creoles where founder effects and genetic drift must have been dramatic considering that the total number of Iberian cattle brought to the Americas was probably less than 1000 [4].

Knowing the genetic history of Creole cattle in the Americas should provide a better understanding of livestock gene flow during the period of discovery and settlement by Iberian colonizers, and the influence that may have resulted from the later introductions of cattle from other European origins and of Zebus from India that begun in the 19th century. In addition, the assessment of genetic diversity and structure of Creole cattle populations is crucial for the development of appropriate management programs aimed at their recognition, conservation and genetic improvement.

The objective of this study was to use neutral genetic markers to retrospectively assess the origins and evolutionary trajectories of American Creole cattle, and investigate the influence that Iberian, European and Zebu breeds may have had on their genetic make-up. The influence of African cattle to the Creole breeds is also discussed, particularly the indirect contribution mediated by their Iberian counterparts. Using a subset of 81 cattle breeds sampled in Europe and the Americas, we show that the majority of the Creole breeds still maintain distinct genetic signatures of Iberian cattle, but some have been admixed with cattle from other geographic regions, mostly of the Zebu type in tropical regions and British and Continental breeds in other parts of the Americas.

## Results

### Genetic Diversity and Breed Differentiation

**Figure 1:**
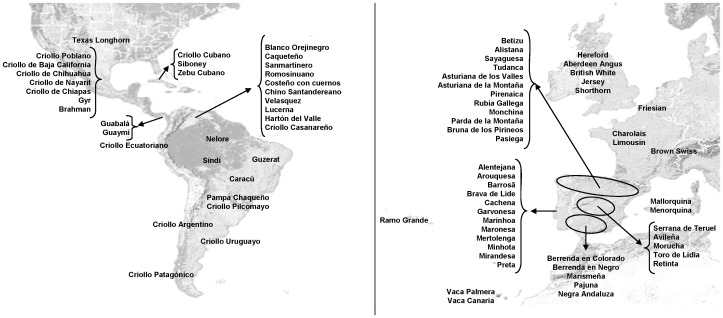
Geographic distribution of the 81 cattle breeds from America and Europe.

A set of 19 microsatellite markers was used to analyze samples of the 81 cattle breeds included in this study (Table S1), which represented the Creole (27 breeds), Iberian (39 breeds), British (5 breeds), Continental European (4 breeds) and Zebu (6 breeds) groups, with the geographical distribution shown in Figure 1.

The microsatellite markers used allowed the detection of a mean number of 6.78±1.88 alleles/locus per breed and 11.93±3.52 alleles/locus per breed group, with global observed and expected heterozygosities of 0.688±0.018 and 0.711±0.025, respectively (Table S2). Taken together, Creole cattle showed the highest mean (14.21±3.74) and effective (4.08±0.57) number of alleles, allelic richness (4.69±0.51), and observed and expected heterozygosities (0.719±0.004 and 0.805±0.014, respectively), when compared with the other breed groups (Table 1).

**Table 1:** Genetic variability estimated for different groups of cattle breeds.

Breed Group	N	Am ± SD	Ae	Ar	Ho ± SD	He ± SD
**Creole**	907	14.21±3.74	4.08	4.69	0.719±0.004	0.805±0.014
**Spanish**	1,199	12.53±3.39	3.85	4.46	0.677±0.003	0.777±0.018
**Portuguese**	675	10.74±3.59	3.62	4.27	0.677±0.004	0.749±0.025
**British**	200	8.89±2.21	3.26	4.41	0.653±0.008	0.754±0.015
**Continental European**	184	9.89±3.45	3.95	4.13	0.720±0.008	0.760±0.020
**Zebu**	168	11.32±3.16	3.31	4.41	0.654±0.009	0.735±0.026
*Mean*		*11.93±3.52*	*3.68±0.34*	*4.40±0.19*	*0.683±0.030*	*0.763±0.025*

Number of individuals sampled (N), mean number of alleles (Am), effective number of alleles (Ae), allelic richness (Ar), observed (H_o_) and expected (H_e_) heterozygosities and their standard deviations (SD). Groups of breeds: ***CREOLE***: Criollo Argentino (CARG), Criollo Patagónico (PAT), Caracú (CAR), Blanco Orejinegro (BON), Caqueteño (CAQ), Criollo Casanareño (CC), Chino Santandereano (CH), Costeño con Cuernos (CCC), Hartón del Valle (HV), Lucerna (LUC), Romosinuano (RMS), Sanmartinero (SM), Velasquez (VEL), Cubano (CUB), Siboney (SIB), Criollo Ecuatoriano (EC), Criollo de Baja California (CBC), Criollo de Chiapas (CHI), Criollo de Chihuahua (CHU), Criollo de Nayarit (CNY), Criollo Poblano (CPO), Guabalá (GUA), Guaymí (GY), Pampa Chaqueño (PA), Criollo Pilcomayo (PIL), Criollo Uruguayo (CUR) and Texas Longhorn (TLH); ***SPANISH***: Alistana (ALS), Asturiana de las Montañas (ASM), Asturiana de los Valles (ASV), Avileña (AVI), Berrenda en Colorado (BC), Berrenda en Negro (BN), Betizu (BET), Bruna de los Pirineos (BRP), Mallorquina (MALL), Menorquina (MEN), Monchina (MON), Morucha (MOR), Marismeña (MAR), Negra Andaluza (NAN), Pajuna (PAJ), Parda de Montaña (PM), Pasiega (PAS), Pirenaica (PIRM), Retinta (RET), Rubia Gallega (RGA), Sayaguesa (SAY), Serrana de Teruel (STE), Toro de Lidia (TL), Tudanca (TUD), Vaca Canaria (VCA) and Vaca Palmera (PAL); ***PORTUGUESE***: Alentejana (ALT), Arouquesa (ARO), Barrosã (BARR), Brava de Lide (BRAV), Cachena (CACH), Garvonesa (GARV), Marinhoa (MARI), Maronesa (MARO), Mertolenga (MERT), Minhota (MINH), Mirandesa (MIRA), Preta (PRET) and Ramo Grande (RG); ***BRITISH***: Aberdeen Angus (AA), British White (BWC), Hereford (HER), Jersey (JER), Shorthorn (SH); ***CONTINENTAL EUROPEAN***: Charolais (CHAR), Friesian (FRI), Limousin (LIM), Brown Swiss (BSW); ***ZEBU***: Brahman (BRH), Gyr (GYR), Guzerat (GUZ), Nelore (NEL), Sindi (SIN), Zebu Cubano (CUZ).

The average F-statistics and their 95% confidence intervals obtained with 10,000 bootstraps over loci were *f* = 0.0326 (0.0231–0.0451), *F* = 0.1360 (0.1250–0.1479) and *<*theta*>* = 0.1069 (0.0977–0.1170). The group means for within-breed deficit in heterozygosity were highest for the Spanish and Zebu breeds (nearly 0.048), and lowest for the Continental European breeds (−0.002±0.026). The Portuguese Mertolenga and Brava, the Spanish Negra Andaluza and the Mexican Criollo Poblano had the highest within-breed F_IS_, with estimates close to 0.11 (Table S2).

Genetic distances among breed pairs, estimated by *<*theta*>* values, ranged from 0.01 to 0.33 (results not shown for individual breeds), with a mean distance of Creoles relative to other breed groups as follows: 0.016 for Spanish, 0.018 for Portuguese, 0.023 for Continental European, 0.033 for British and 0.095 for Zebu breeds (Table 2). The estimated number of migrants, i.e., the number of individuals exchanged between populations per generation that would balance the diversifying effect of genetic drift, was highest for the Creole, Spanish and Portuguese pairs, while the Zebu had the lowest number of migrants relative to all the other groups.

**Table 2:** Genetic distances among breed groups.

	CRE	SP	PT	BR	EU	ZEB
**CRE**		0.016	0.018	0.033	0.023	0.095
**SP**	15.80	–	0.013	0.036	0.020	0.135
**PT**	13.78	18.51	–	0.041	0.027	0.143
**BR**	7.43	6.66	5.79	–	0.032	0.159
**EU**	10.43	11.96	9.12	7.57	–	0.156
**ZEB**	2.38	1.60	1.50	1.33	1.35	–

Genetic distances estimated by Weir and Cockerham *<*theta*>* (above diagonal) and corresponding number of migrants (below diagonal). Breed groups: CRE – Creole; SP – Spanish; PT – Portuguese; BR – British; EU – Continental European; ZEB –Zebu. See Table 1 for the definition of breeds included in each group.

**Figure 2:**
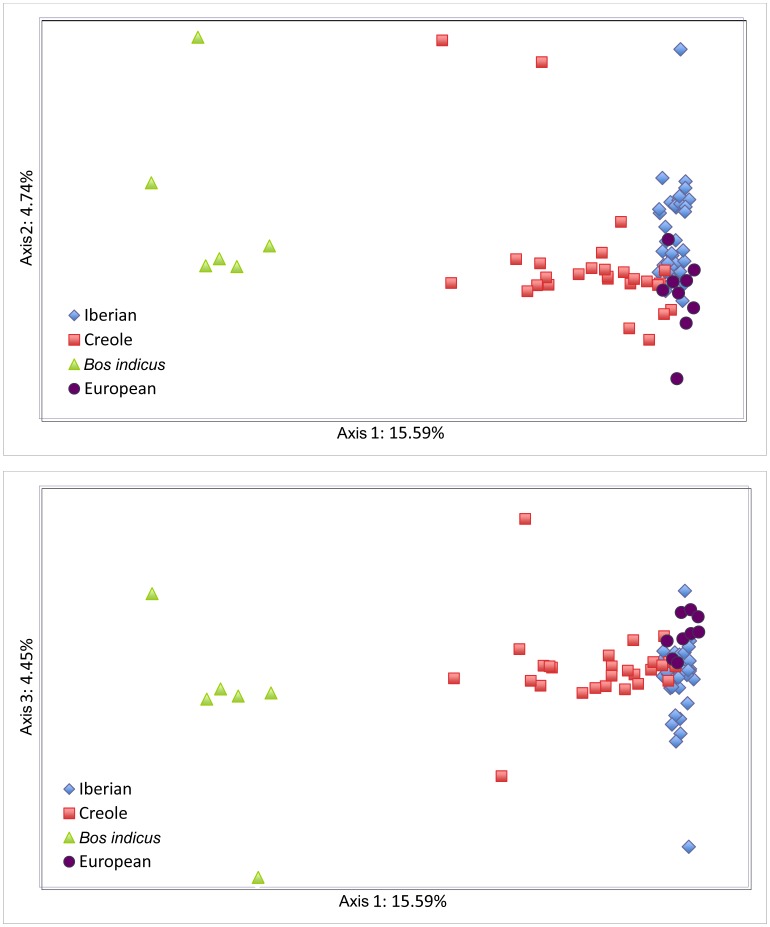
Graphical representation of the three first axes from the factorial correspondence analysis of the 81 cattle breeds from America and Europe.

The results from the Factorial Correspondence Analysis (Figure 2) indicate that the first three FCA axes explain <25% of the variability. The first axis accounts for about 16% of the variability and separates *Bos indicus* from the remaining breeds. The second component, which accounts only for 5% of the variability, essentially separates the Iberian and the European breeds, while the third component accounts for a small 4% of the total variability and allows for some splitting among breeds in the same genetic or geographical group. The Creoles occupy a more central position in the graph and, depending on the breed considered, they have a closer proximity to the Iberian, European or Indicine clusters, reflecting the influence that these groups have had in their genetic make-up.

The AMOVA results indicated that the highest percentage of variation among groups (11.4%, P<0.001) was found when breeds deriving from *B. taurus* and *B. indicus* were compared (results not shown). When the genetic differentiation of Creoles relative to Iberian, European and Zebu breeds was considered, the largest amount of variability was found between Creole and Zebu populations (9.15%, P<0.001), and the lowest between the Creole and Iberian breeds (1.09%, P<0.001).

### Population Genetic Structure

**Figure 3:**
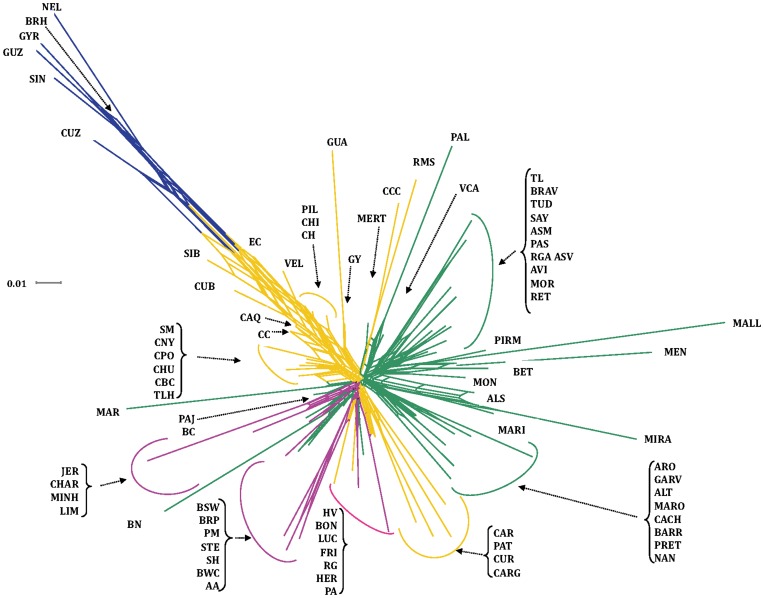
Neighbor-net dendrogram constructed from the Reynolds genetic distances among 81 cattle breeds. Yellow: Creole; Green: Iberian; Pink: British and Continental European; Blue: Indian Zebu. ***SPANISH***. Betizu (BET), Toro de Lidia (TL), Menorquina (MEN), Alistana (ALS), Sayaguesa (SAY), Tudanca (TUD), Asturiana de los Valles (ASV), Asturiana de las Montañas (ASM), Retinta (RET), Morucha (MOR), Avileña (AVI), Pirenaica (PIRM), Rubia Gallega (RGA), Mallorquina (MALL), Monchina (MON), Serrana de Teruel (STE), Parda de Montaña (PM), Bruna de los Pirineos (BRP), Pasiega (PAS), Berrenda en Colorado (BC), Berrenda en Negro (BN), Marismeña (MAR), Pajuna (PAJ), Negra Andaluza (NAN), Vaca Canaria (VCA), Vaca Palmera (PAL); ***PORTUGUESE***. Alentejana (ALT), Arouquesa (ARO), Barrosã (BARR), Brava de Lide (BRAV), Cachena (CACH), Garvonesa (GARV), Marinhoa (MARI), Maronesa (MARO), Mertolenga (MERT), Minhota (MINH), Mirandesa (MIRA), Preta (PRET), Ramo Grande (RG); ***CREOLE***. Guabalá (GUA), Guaymí (GY), Texas Longhorn (TLH), Criollo Poblano (CPO), Criollo de Baja California (CBC), Criollo de Chihuahua (CHU), Criollo de Nayarit (CNY), Criollo de Chiapas (CHI), Blanco Orejinegro (BON), Caqueteño (CAQ), Sanmartinero (SM), Romosinuano (RMS), Costeño con Cuernos (CCC), Chino Santandereano (CH), Velasquez (VEL), Lucerna (LUC), Hartón del Valle (HV), Criollo Casanareño (CC), Criollo Ecuatoriano (EC), Criollo Uruguayo (CUR), Pampa Chaqueño (PA), Criollo Pilcomayo (PIL), Criollo Argentino (CARG), Criollo Patagónico (PAT), Caracú (CAR), Cubano (CUB), Siboney (SIB); ***ZEBU***: Gyr (GYR), Brahman (BRH), Sindi (SIN), Guzerat (GUZ), Nelore (NEL), Zebu Cubano (CUZ); Other ***EUROPEAN.*** Friesian (FRI), Hereford (HER), Brown Swiss (BSW), Aberdeen Angus (AA), British White (BWC), Charolais (CHAR), Jersey (JER), Limousin (LIM), Shorthorn (SH).

The Neighbor-net built with the Reynolds distances (Figure 3) supports the existence of two major clusters, corresponding to *B. indicus* and *B. taurus* breeds, with several Creole breeds grouped in the *B. indicus* cluster, which is interpreted as a sign of Zebu influence in their genetic make-up. These included the Creoles from Cuba and Ecuador, the Pilcomayo from Paraguay, the Criollo de Chiapas from Mexico and some Creole breeds from Colombia (Chino Santandereano, Caqueteño and Criollo Casanareño). Among the Creoles showing a residual zebu influence, the Texas Longhorn and the majority of the Mexican Creoles were closely clustered at the centre of the dendrogram, displaying a common origin with the Spanish Marismeña. Another Creole cluster, made-up by the Romosinuano and Costeño con Cuernos from Colombia and, to a lesser extent, the two breeds from Panama, showed a common origin with the breeds from the Canary Islands and the Portuguese Mertolenga. The Creoles from Argentina and Uruguay and the Caracu from Brasil formed an independent cluster at the center of the dendrogram, with a weak relationship with British breeds. On the other hand, the Pampa Chaqueño from Paraguay and the Harton del Valle, Lucerna and Blanco Orejinegro from Colombia showed a clear influence of British breeds.

Among Iberian breeds, several different clusters could be identified, such that nearly all Portuguese breeds grouped together, with the major exception of the Mirandesa, which clustered with breeds with a close geographic distribution, both in Portugal and Spain. Another cluster corresponded to the breeds from the Balearic Islands, which grouped with a few breeds from northern Spain, while the majority of the Spanish breeds clustered together. A distinct cluster corresponded to the breeds from the Canary Islands, which also included the Portuguese Mertolenga. Two Spanish breeds were isolated from the remaining clusters, i.e., the Marismeña and the Berrenda en Negro. The remaining Iberian breeds (Minhota and Ramo Grande from Portugal, Bruna de los Pirineos, Serrana de Teruel and Parda de Montaña from Spain) were close to Continental European breeds, indicating some admixture with these breeds.

The Bayesian clustering model-based method [20] allowed for assessment of the genetic structure and admixture among breeds. When the number of ancestral populations varied from K = 2 to 81, the largest change in the log of the likelihood function (ΔK) was when K = 71 (Figure S1).

**Figure 4:**
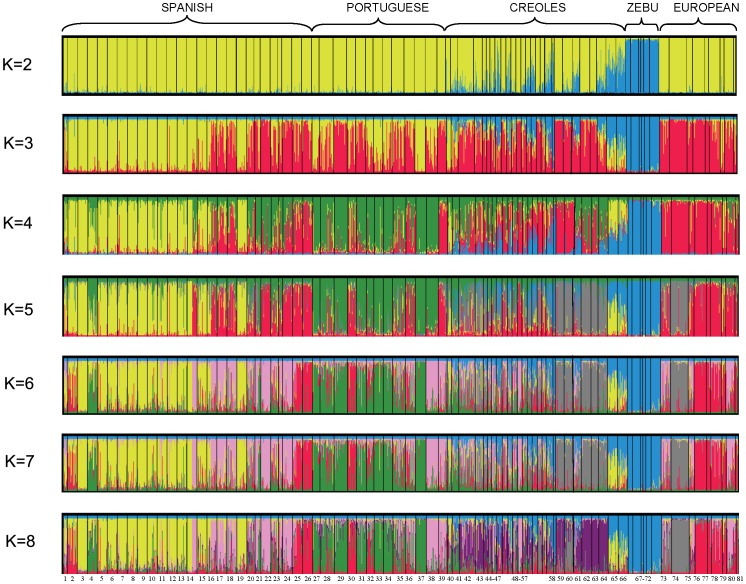
Population structure of 81 cattle breeds based on 19 microsatellite loci using STRUCTURE. Graphical representation of individual genotype membership coefficients (q) when K = 2 to K = 8. Each animal is represented by a single vertical line divided into K colours, where K is the number of clusters assumed and the coloured segment shows the individual’s estimated membership proportions in that cluster. Black lines separate the populations. ***SPANISH***. 1: Betizu (BET), 2: Toro de Lidia (TL), 3: Menorquina (MEN), 4: Alistana (ALS), 5: Sayaguesa (SAY), 6: Tudanca (TUD), 7: Asturiana de los Valles (ASV), 8: Asturiana de las Montañas (ASM), 9: Retinta (RET), 10: Morucha (MOR), 11: Avileña (AVI), 12: Pirenaica (PIRM), 13: Rubia Gallega (RGA), 14: Mallorquina (MALL), 15: Monchina (MON), 16: Serrana de Teruel (STE), 17: Parda de Montaña (PM), 18: Bruna de los Pirineos (BRP), 19: Pasiega (PAS), 20: Berrenda en Colorado (BC), 21: Berrenda en Negro (BN), 22: Marismeña (MAR), 23: Pajuna (PAJ), 24: Negra Andaluza (NAN), 25: Vaca Canaria (VCA), 26: Vaca Palmera (PAL); ***PORTUGUESE***. 27: Alentejana (ALT), 28: Arouquesa (ARO), 29: Barrosã (BARR), 30: Brava de Lide (BRAV), 31: Cachena (CACH), 32: Garvonesa (GARV), 33: Marinhoa (MARI), 34: Maronesa (MARO), 35: Mertolenga (MERT), 36: Minhota (MINH), 37: Mirandesa (MIRA), 38: Preta (PRET), 39: Ramo Grande (RG); ***CREOLE***. 40: Guabalá (GUA), 41: Guaymí (GY), 42: Texas Longhorn (TLH), 43: Criollo Poblano (CPO), 44: Criollo de Baja California (CBC), 45: Criollo de Chihuahua (CHU), 46: Criollo de Nayarit (CNY), 47: Criollo de Chiapas (CHI), 48: Blanco Orejinegro (BON), 49: Caqueteño (CAQ), 50: Sanmartinero (SM), 51: Romosinuano (RMS), 52: Costeño con Cuernos (CCC), 53: Chino Santandereano (CH), 54: Velasquez (VEL), 55: Lucerna (LUC), 56: Hartón del Valle (HV), 57: Criollo Casanareño (CC), 58: Criollo Ecuatoriano (EC), 59: Criollo Uruguayo (CUR), 60: Pampa Chaqueño (PA), 61: Criollo Pilcomayo (PIL), 62: Criollo Argentino (CARG), 63: Criollo Patagónico (PAT), 64: Caracú (CAR), 65: Cubano (CUB), 66: Siboney (SIB); ***ZEBU***: 67: Gyr (GYR), 68: Brahman (BRH), 69: Sindi (SIN), 70: Guzerat (GUZ), 71 Nelore (NEL), 72: Zebu Cubano (CUZ); ***BRITISH AND CONTINENTAL*** ** ***EUROPEAN.*** 73: Friesian (FRI), 74: Hereford (HER), 75: Brown Swiss (BSW), 76: Aberdeen Angus (AA), 77: British White (BWC), 78: Charolais (CHAR), 79: Jersey (JER), 80: Limousin (LIM), 81: Shorthorn (SH).

The results for K = 2 (Figure 4) indicate a clear separation between *B. indicus* and *B. taurus* breeds. Moreover, these results confirm the admixture of Zebu with some of the Creole breeds, especially Siboney, Criollo Cubano, Criollo Ecuatoriano, Pilcomayo, Casanareño and Velasquez, while other breeds, such as the Creoles from Argentina and Uruguay, and the Romosinuano, Sanmartinero and Blanco Orejinegro from Colombia, show minor signs of Zebu admixture.

When three ancestral populations were inferred, the breeds from Northern Spain and the Portuguese Mirandesa and Marinhoa, separated from the remaining *B. taurus* breeds, whereas the other breeds from Portugal and Southern Spain remained clustered with the Creole breeds. As the number of inferred ancestral populations increased, admixture among breeds became more apparent, but some Creole breeds, such as the two Argentinean and the Uruguayan Creoles, Caracú from Brazil, Texas Longhorn, Creoles of Baja California and Poblano from Mexico, and the Romosinuano and Costeño con Cuernos from Colombia, remained very homogeneous at K = 8. For the 81 cattle breeds analysed, the most likely number of inferred ancestral populations was K = 71 (Figure S2), as assessed by the method of Evanno et al. (2005). The computed individual membership coefficients resulted in about 60–70% of the individuals classified within their source ancestral population, assuming a threshold of *q*>0.8. The Zebu breeds Brahman, Guzerat, Gyr, Nelore and Sindi grouped together in the same cluster with values of *q* around 0.700 while Cuban Zebu grouped in the same cluster with Criollo Cubano. The Mexican Creoles, with the exception of the Criollo de Chiapas, clustered together, in the same way that the Creoles from Colombia Chino Santandereano, Velasquez, Casanare and Caqueteño formed a unique cluster, although with low *q* values (Table S3).

### Ancestral Genetic Contributions to Creole Cattle

**Figure 5:**
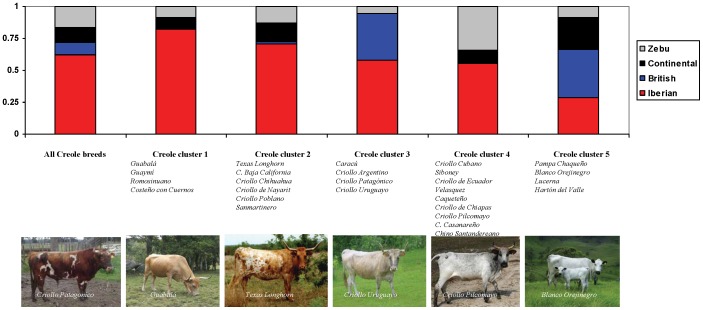
Genetic contributions from Iberian, British, Continental European and Zebu breeds to Creole cattle. Graphical representation of maximum-likelihood estimates of proportional genetic contributions form some groups of breeds to Creole cattle considered as a whole or grouped in five different clusters. The Creole breeds included in each cluster are listed below, and photos of animals representative of each cluster are also shown.

The estimated genetic contributions of each potential ancestral breed group (Iberian, British, Continental European and Zebu) to Creole cattle are shown in Figure 5 and Table S4, as computed by the likelihood estimation of admixture proportions developed by Wang [21], and implemented by the LEADMIX software. The admixture estimates indicate that, for the Creole cattle considered as a single group, Iberian cattle contributed nearly 62% to the genetic pool, Zebu breeds contributed about 17% and Continental European and British breeds about 10% each.

The Neighbor-net indicated the existence of various Creole clusters, which is also supported by the analysis carried-out with STRUCTURE. These clusters likely reflect different contributions from the ancestral genetic groups to the current genetic pool of Creoles. Therefore, a similar analysis of estimated genetic contributions was carried out with LEADMIX for each of the five identified Creole clusters, as shown in Figure 5. These analyses revealed clear differences among the five clusters in the relative contributions of the four parental genetic groups. The Creoles from Panama, Mexico, United States and some Colombian breeds (Clusters 1 and 2) showed the strongest Iberian influence, with nearly 70 to 80% of the genetic pool contributed by Iberian breeds, with the remaining contributions corresponding to Continental and Zebu breeds, in about equal proportions. The Creoles from the southern region of the Americas (Cluster 3) had an important influence of about 60% from Iberian breeds, but also showed influence from British cattle. Cluster 4, which corresponds to Creole breeds widely dispersed in tropical areas, showed an important contribution from Zebu breeds, even though the Iberian contribution was still predominant. The Paraguayan and Colombian breeds included in Cluster 5 show a major influence of British and Continental breeds, with a smaller but still detectable contribution of Iberian cattle.

## Discussion

The genetic relationships between Creole cattle and their presumed ancestral sources remain largely unexplored. Estimates of genetic diversity and population structure have been previously reported for some Creole populations [9], [22–26], for Iberian cattle [13], [27]–[30], Zebu breeds [31], [32] and European cattle [10], [33]. Moreover, mtDNA and Y-chromosome markers were used to investigate the origins of Creole cattle [34]–[38], but their genetic relationship with other cattle breeds which could have influenced them remained unclear.

Our study combines several data sets that cover a wide range of Creole, European and Indicine cattle populations, thus providing a more comprehensive insight about the genetic influences that Creole breeds received since the arrival of the first Iberian cattle in the American continents in the late 1400’s.

Of the total genetic variability, nearly 11% is explained by breed differences, which is slightly higher than what has been reported for other cattle breeds around the world, generally in the range of 7 to 9% [10], [28], [38]. This could be justified by the inclusion in this study of cattle breeds representing the two well differentiated phylogenetic groups of *B. indicus* and *B. taurus* [39].

The high genetic variability found in Creole cattle, even in populations considered as endangered, might reflect recent contributions of cattle from different origins, which are known to have been admixed with some Creole populations over the last century [40], [41]. This result is in agreement with the analysis of mtDNA sequences and Y haplotypes, which have shown the genetic heterogeneity of Creole cattle, in which signatures of Iberian, European and Indian cattle are detected, and the direct influence of African cattle has also been claimed [35], [37], [42].

Recently, Gautier and Naves (2011) [43] used a high-density panel of SNPs to study genetic influences in Creole cattle from Guadeloupe and reported evidence of a direct African ancestry in this breed. In our study, no African samples were included, but some results may be interpreted as indicating a possible African influence on some Creole populations. For example, allele 123 in the BM2113 locus has previously been associated with West African taurine cattle [44], and is present at high frequencies in some Creole populations such as Caqueteño, Sanmartinero and Pilcomayo. On the other hand, allele 143 at the same locus has been considered an indication of African Zebu influence [44], and it is present in Caracú, which could be regarded as further evidence of African influence on Creoles. It is not clear, however, if these African signatures correspond to a direct contribution of African cattle to Creoles, or rather to an indirect influence through Iberian cattle, given that some Iberian breeds in our study also have a high frequency of alleles considered to be African-specific [30]. Also, it has been suggested that the Zebu influence detected in Creoles may be in part due to *B. indicus* cattle imported from Africa during the colonial period [43]. Studies where mtDNA sequence variation was analyzed have confirmed the presence of African matrilines among Creoles [37], [42], [45], and Y-haplotypes have also revealed a possible West African signature in Creole cattle [35]. Overall, our results provide strong support to the conclusion of an Iberian influence on Creole cattle, but are not so clear in elucidating the possibility of a direct African influence on the different Creole groups. Further studies are necessary, covering a broad sample of African cattle breeds and a combination of different genetic markers, to clarify the African influence on Creoles.

The degree of genetic differentiation among all breeds studied indicates relatively low levels of gene flow and some level of reproductive isolation among most Creole breeds, probably as a result of geographic separation and differentiation. Taken together, Creole breeds differ more from *B. indicus* than from the remaining *B. taurus* breeds, with the lowest levels of differentiation for the Creole-Spanish and Creole-Portuguese pairs. Our results indicate that Creole cattle retain genetic signatures of their Iberian ancestry, in agreement with previous studies based on monoparental genetic markers [35].

The F-statistics, AMOVA and Factorial Correspondence Analysis results confirmed the closer proximity of Creoles to Iberian breeds, and their much larger differentiation from Zebu cattle. Nevertheless, Indian Zebus have had an important influence on some Creole breeds, as is clear from the Reynolds Neighbor-net, the Bayesian approach adopted by STRUCTURE and the maximum-likelihood estimation of genetic contributions from parental populations carried-out with LEADMIX. However, the majority of Creoles seem to have been largely unaffected by the introduction of Zebus into South America in the 19th and 20th centuries. In general, Creole breeds from tropical areas (Siboney, Criollo Cubano, Criollo Ecuatorino, Criollo de Chiapas and some Colombian breeds), showed the highest degree of admixture with Zebu, but this influence extended as far south as the Criollo Pilcomayo from Paraguay.

The present study indicates that the influence of Iberian cattle was mostly due to the breeds from Portugal and Southern Spain, which had a closer relationship with Creoles, as detected in the Bayesian analysis with STRUCTURE. In the period when Central and South America were settled, cattle breeds from Portugal and Spain were probably not very distinct from each other, given that the major period of breed formation started in the late 18th century [46]. Also, Portugal and Spain were united in 1580 under Philip II, King of Spain, and kept together until 1640, and this corresponded to a period of major livestock shipments from the Iberian Peninsula to South America [48]. It is also known that the vast majority of the expeditions to South America departed from Lisbon (central Portugal) or from Cádiz and Seville (Southern Spain), which would explain the closer relationship of Creoles with cattle breeds from these regions. The shipment of cattle directly from Africa to the Americas could have occurred following slave routes and from intermediate ports in the Atlantic Islands.

The establishment of Iberian livestock in the “New World” followed two different migration routes, depending on the predominance of colonizers being Spanish or Portuguese. The first arrival of cattle was in 1493, when Columbus brought animals from Spain to the Caribbean Islands in his second trip, and the presence of hundreds of animals in Cuba was reported four decades later [3]. Cattle from the Caribbean Islands, e.g., Cuban Creole, could thus be considered a remnant of the first cattle brought from Spain, but it is widely recognized that Zebu cattle have been extensively used in crossbreeding with Caribbean cattle over the last century [4], [40]; it is, therefore, difficult to identify a specific breed from this area as a good representative of the early Iberian stock.

From the Caribbean Islands, where animals were stocked and bred, cattle were brought to North America through the Mexican port of Veracruz, and from there they expanded throughout Mexico and towards the region corresponding to Texas. The Texas Longhorn and the different Mexican Creole populations could be regarded as representing this path of cattle dispersion.

An additional route of dispersion of animals from the Caribbean was into Central America through the ports of Panama or directly into the northern part of South America, through the port of Santa Marta in Colombia [47]. From these places, they were then distributed to the Northern Peruvian Vice Kingdom (today, Colombia, Ecuador and Peru). The Panamanian and some of the Colombian breeds could be considered as representatives of this path of cattle dispersion.

The Rio de la Plata was an important route of distribution of Iberian cattle in Southern South America, often disseminated by Jesuit missions which had a strong influence in this area [48]. From Rio de la Plata cattle dispersed through most of South America, from Patagonia up to the southern part of the Peruvian Vice Kingdom. In these areas, the Spanish path of dispersion was probably mixed with the Portuguese route, in which cattle were shipped from Portuguese ports into the Cape Verde Archipelago and from there to the Captaincies on the Brazilian coast, mainly to Pernambuco and Bahia in the North, and São Vicente (near Rio de Janeiro) in the South [48]. The southern part of Brazil, Uruguay and Argentina have milder climate, and the Zebu influence was probably less severe than in tropical Central America. Several breeds from Southern South America included in our studies confirm lower levels of Zebu admixture, including the Argentinean, Patagonian and Uruguayan Creoles as well as the Brazilian Caracú, even though the latter may have suffered some Zebu introgression in the past [35]. Nevertheless, it is known that British cattle, especially Hereford and Angus, were introduced and widely expanded in this region in the mid-19th century, and could have had some influence on Creole populations.

Our study provides evidence that Creole breeds still show important influences of Iberian cattle, which contributed with nearly two-thirds of the Creole genetic pool analyzed. Our results further indicate that large genetic differences exist among Creole sub-populations, reflecting the effects of genetic drift as well as the introduction of other breeds through the years after the initial arrival of Iberian cattle. The Neighbor-net representation of the pairwise Reynolds genetic distances generally supports the existence of five Creole clusters, which may correspond to different paths of cattle dispersion into the Americas or, in some cases, perhaps to a more recent admixture of germplasm from other breeds.

The first cluster corresponds to the Panamanian (Guabalá and Guaymi) and some of the Colombian breeds (Costeño con Cuernos and Romosinuano), and could represent the first route of cattle introduction into Central and South America from the Caribbean Islands, as the flow of ships between Panama and Colombia was very important in the 16th century [48]. This cluster presents the highest Iberian and possibly African influences, with minor contributions from Continental and Zebu breeds. Interestingly, a common origin was detected between the Costeño con Cuernos and Romosinuano from Colombia and the breeds from the Canary islands, lending support to the important role played by this archipelago as a point of shipment and reload of animals taken to the Americas [3], [48].

The second cluster is represented by most of the Mexican breeds, the Colombian Sanmartinero and the Texas Longhorn, which are near the centre of the radial net, and could correspond to one of the first paths of cattle dispersion into Central and North America. This cluster still shows a major signature of Iberian cattle, with minor influences from Continental and Zebu breeds. A very interesting result in our study was the detection of a common origin shared by this group of breeds and the Spanish Marismeña. It is generally believed that, since Columbian times, the Marismeña has been kept since Colombian times in semi-feral conditions in a natural park near the original point of departure of Spanish sailors [49]. Our results confirm that it could represent a remnant of the animals taken to the Americas in the early period of settlement.

The third cluster contains the Brazilian Caracú and the Argentinean, Patagonian and Uruguayan Creoles. This group is likely a representative of the Rio de la Plata and Brazilian routes of colonization and cattle flow into South America. Even though the Iberian contribution is very clear in this group, the later introduction of British cattle in the region probably resulted in some admixing, which is now detectable in the genetic pool of this cluster, particularly in the Uruguayan Creoles.

The fourth cluster includes breeds with a widely dispersed geographical distribution, such as the Creoles from Cuba and Ecuador, the Velasquez, Caqueteño, Casanareño and Chino Santandereño from Colombia, the Chiapas Creole from Southern Mexico and the Pilcomayo from Paraguay. All these breeds still show a major Iberian contribution but also a strong Zebu influence, confirming the impact of *B. indicus* on the genetic make-up of Creole cattle in many tropical areas during the last century.

The fifth cluster includes three Colombian breeds (Blanco Orejinegro, Lucerna and Harton del Valle) and the Paraguayan Pampa Chaqueño. This cluster shows some proximity with British and Continental breeds, and a minor, but still detectable, representation of the Iberian contribution.

Our results generally support the historical descriptions of cattle introduction and dispersion throughout the Americas [3], [48]. Nearly 500 years after their arrival, strong genetic signatures of Iberian cattle are still present in Creoles. The major ancestral contributions are from breeds of Southern Spain and Portugal, in agreement with the historical ports of departure of ships sailing towards the Western Hemisphere. Furthermore, the role of the Canary Islands in the flow of cattle to the Americas was confirmed.

Even though the term “Creole” has been used since early colonial times in Latin America in reference to both people and animals born in the newly-discovered land from parents of Iberian origin [7], it is clear that there is more genetic variability among Creole cattle in comparison to breeds from other geographic regions. This diversity results from differential genetic contributions from several parental populations, genetic drift and some admixing with other breeds over time. Based on the information derived from our study, it is possible to summarize the gene flow that gave origin to the different Creole populations, confirming the influence of Iberian, British, Continental and Zebu breeds.

The major feature that should be retained is the predominant influence of Iberian cattle on Creoles still present today. Signatures of African cattle are also represented in many Creole breeds, and which can result from either direct contributions or indirect influences through Iberian cattle. Evidence of admixing with British breeds is visible in some Colombian and Paraguayan breeds and, to a lesser extent, in Creoles from southern South America. Creoles from tropical areas, especially those from the Caribbean, show clear admixture with Zebu, which contribute high tolerance to hot and humid climates, and resistance to parasites. Some Creole populations still show a close proximity to their distant Iberian ancestors, and efforts should be made to avoid their extinction or further genetic erosion through admixture with other breeds, which would compromise five centuries of selective adaptation to environmental conditions which range from the deserts of Texas and Mexico, to the mountains of Patagonia.

Overall, our study indicates that: 1) several centuries after the introduction of Iberian cattle into America, Creole breeds still show strong and predominant signatures of Iberian influence; 2) Creole breeds differ widely from each other, both in their genetic structure and in the genetic influences received from other breeds; 3) in some Creole breeds, especially those from tropical regions, the impact of *B. indicus* is very clear, even though the Iberian influence is still prevalent; 4) a few Creole breeds from Colombia and Paraguay have a major influence from British and Continental breeds.

This study provides significant genetic information about cattle populations in the Americas that are remnants of historical colonization. Our findings reveal the evolutionary trajectories of cattle in close association with human dispersal and confirm Creoles as legitimate representatives of cattle from the discoveries. Furthermore, our results provide the means to identify the Creole breeds with different genetic signatures, which will be useful for the development of global and local conservation of cattle genetic diversity.

## Materials and Methods

### Samples

The study included biological samples of 3,333 animals representing 81 cattle breeds from 12 different countries (Table S1). The origin of the breeds studied (Figure 1) was either Creole (a comprehensive sample of 27 breeds, representing a wide range of Creole cattle, from North America to Patagonia), Iberian (39 native breeds from Portugal and Spain, including 3 breeds from the Atlantic Islands), European breeds (9 *B. taurus* breeds from the British Isles and Continental Europe which have been widely used throughout the world) and Zebu breeds (6 breeds representing the *B. indicus* group).

Semen samples were obtained from germplasm banks. Blood and hair root samples were collected by qualified veterinarians through their routine practice, in the framework of oficial programs aimed at the identification, health control and parentage confirmation of the breeds and populations included in our study. Therefore, the legal restrictions defined in “Spanish Law 32/2007 of November 7, on the care of animals in their husbandry, transportation, testing and sacrifice” do not apply, as they are waved in the case of non-experimental procedures and routine veterinary practices with livestock species, in Article 3d of the above-mentioned Law.

### Molecular Markers

Six laboratories were involved in this study (Universidad de Córdoba, Universidad Complutense de Madrid and Universidad de Zaragoza from Spain, Instituto Nacional dos Recursos Biológicos from Portugal, University of California in Davis from the United States of America, and Universidad Nacional de Colombia in Palmira from Colombia).

A common set of 19 microsatellites were selected from a panel of 30 markers recommended for genetic diversity studies by the International Society for Animal Genetics (ISAG) / Food and Agriculture Organization of the United Nations (FAO) working group [50]: *BM1818, BM1824, BM2113, CSRM60, CSSM66, ETH3, ETH10, ETH185, ETH225, HAUT27, HEL9, ILSTS006, INRA032, INRA063, MM12, SPS115, TGLA53, TGLA122* and *TGLA227*.

### DNA Amplification, and Genotyping

Genomic DNA was extracted using procedures previously described [13], [29], [51] The 19 microsatellite markers were amplified in multiplex polymerase chain reactions (PCRs) using fluorescence-labelled primers [13]. PCR products were separated by electrophoresis on ABI instruments (3730, 3130 and 377XL, Applied Biosystems, Foster City, CA) according to manufacturer recommendations and allele sizing was accomplished by using the internal size standards GeneScan™-500 LIZ™ and GeneScan-400HD ROX (Applied Biosystems, Warrington, UK).

Allele nomenclature was standardized following a former European research project on cattle genetic diversity (EU RESGEN CT 98–118, for further details on the project outcome Dr J. A. Lenstra has to be contacted: J.A.Lenstra@uu.nl). To assure compatibility of results from different equipments and laboratories, a total of 30 samples representing the entire allele range for this set of markers was exchanged and genotyped in all laboratories. Allele sizing was standardized across laboratories based on these reference samples. Moreover, reference samples (2) were included in each assay to control for variation between electrophoresis.

### Statistical Analysis

Data used in this paper have been archived at Dryad (www.datadryad.org): doi:10.5061/dryad.17 gk0.

Mean number of alleles (Am), observed (Ho) and unbiased expected (He) estimates of gene diversity [52] and their standard deviations were obtained with the MICROSATELLITE TOOLKIT software [53]. Distribution of genetic variability within and between breeds was studied by analysing F-statistics [54] as implemented in GENETIX v4.04 [55]. The within-breed inbreeding coefficient (F_IS_) was calculated with a 95% confidence interval obtained by 10000 bootstraps across loci. The effective number of alleles (Ae) and allelic richness (Ar) over all loci per breed were calculated with POPGENE [56] and FSTAT v. 2.9.3 [57], respectively. Deviations from Hardy–Weinberg equilibrium (HWE) were assessed with GENEPOP v. 3.4 software [58]. Both global tests across populations and loci as well as tests per locus per breed were carried-out using the method of Guo & Thompson (1992) [59] and the p-values were obtained using a Markov chain of 10000 dememorization steps, 100 batches, and 5000 iterations.

After defining groups of breeds by geographic origin and ancestry (i.e., Creole, Spanish, Portuguese, British, Continental European and Zebu), a hierarchical analysis of variance was performed to partition the total genetic variance into components due to inter-individual and inter-breed differences. Variance components were used to compute fixation indices and their significance was tested using a non-parametric permutation approach [60]. Computations were carried out using the AMOVA (Analysis of Molecular Variance) module of ARLEQUIN 3.01 [61].

Genetic divergence among breeds was estimated by calculating the Reynolds distances [62] with the POPULATIONS software [63]. A Neighbor-net was constructed with the Reynolds distances using SPLITSTREE 4 [64] to graphically represent the relationships between breeds and to depict evidence of admixture.

Factorial Correspondence Analysis [65] was performed using the function “AFC 3D sur populations” of GENETIX v4.04.

The STRUCTURE v.2.1 software [20] was used to investigate the genetic structure of the 81 cattle populations, in order to identify population substructure and admixture, and to assign individuals to populations. Runs of 10^6^ iterations after a burn-in period of 300000 iterations were performed for each K to determine the most probable number of clusters, as inferred from the observed genotypic data. Ten independent simulations for K equal to 2 to 81 were performed, and the method of Evanno *et al.* (2005) [66] was used to identify the most probable K, by determining the modal distribution of ΔK. The DISTRUCT v.1.1 software [67] was used to obtain a graphical display of individual membership coefficients in each ancestral population, considering the run with the highest posterior probability of the data at each K value.

In order to assess the relative genetic contributions of breeds from different regions (Iberian, British, Continental European and Zebu) in the development of Creoles, a maximum likelihood estimation of admixture proportions was carried out with the LEADMIX software, following the principles described by Wang (2003) [21]. These analyses were conducted for the full group of Creoles, and for five different Creole clusters, as revealed by the Reynolds genetic distances and the corresponding dendrogram.

## Supporting Information

Figure S1 **Graphical representation of ΔK values for K = 2 to K = 81.** Representation for 81 Cattle breeds based on STRUCTURE results following Evanno criterion.
(TIF)

Figure S2 **Population structure of 81 cattle breeds using STRUCTURE when K = 71.** Graphical representation of individual genotype membership coefficients (q) when K = 71. Each animal is represented by a single vertical line divided into 71 coloured segments using only 6 colours showing the individual’s estimated membership proportions in that cluster. 1: Betizu (BET), 2: Toro de Lidia (TL), 3: Menorquina (MEN), 4: Alistana (ALS), 5: Sayaguesa (SAY), 6: Tudanca (TUD), 7: Asturiana de los Valles (ASV), 8: Asturiana de las Montañas (ASM), 9: Retinta (RET), 10: Morucha (MOR), 11: Avileña (AVI), 12: Pirenaica (PIRM), 13: Rubia Gallega (RGA), 14: Mallorquina (MALL), 15: Monchina (MON), 16: Serrana de Teruel (STE), 17: Parda de Montaña (PM), 18: Bruna de los Pirineos (BRP), 19: Pasiega (PAS), 20: Berrenda en Colorado (BC), 21: Berrenda en Negro (BN), 22: Marismeña (MAR), 23: Pajuna (PAJ), 24: Negra Andaluza (NAN), 25: Vaca Canaria (VCA), 26: Vaca Palmera (PAL), 27: Alentejana (ALT), 28: Arouquesa (ARO), 29: Barrosã (BARR), 30: Brava de Lide (BRAV), 31: Cachena (CACH), 32: Garvonesa (GARV), 33: Marinhoa (MARI), 34: Maronesa (MARO), 35: Mertolenga (MERT), 36: Minhota (MINH), 37: Mirandesa (MIRA), 38: Preta (PRET), 39: Ramo Grande (RG); ***CREOLE***. 40: Guabalá (GUA), 41: Guaymí (GY), 42: Texas Longhorn (TLH), 43: Criollo Poblano (CPO), 44: Criollo de Baja California (CBC), 45: Criollo de Chihuahua (CHU), 46: Criollo de Nayarit (CNY), 47: Criollo de Chiapas (CHI), 48: Blanco Orejinegro (BON), 49: Caqueteño (CAQ), 50: Sanmartinero (SM), 51: Romosinuano (RMS), 52: Costeño con Cuernos (CCC), 53: Chino Santandereano (CH), 54: Velasquez (VEL), 55: Lucerna (LUC), 56: Hartón del Valle (HV), 57: Criollo Casanareño (CC), 58: Criollo Ecuatoriano (EC), 59: Criollo Uruguayo (CUR), 60: Pampa Chaqueño (PA), 61: Criollo Pilcomayo (PIL), 62: Criollo Argentino (CARG), 63: Criollo Patagónico (PAT), 64: Caracú (CAR), 65: Cubano (CUB), 66: Siboney (SIB); ***ZEBU***: 67: Gyr (GYR), 68: Brahman (BRH), 69: Sindi (SIN), 70: Guzerat (GUZ), 71 Nelore (NEL), 72: Zebu Cubano (CUZ), 73: Friesian (FRI), 74: Hereford (HER), 75: Brown Swiss (BSW), 76: Aberdeen Angus (AA), 77: British White (BWC), 78: Charolais (CHAR), 79: Jersey (JER), 80: Limousin (LIM), 81: Shorthorn (SH).
(TIF)

Table S1 **Breeds, samples and origins.** Breed names, acronyms (Acron.), sample sizes (N), sample type, breed type, genetic group (GG), country of sampling and region of origin (Reg) of the 81 breeds included in this study.
(PDF)

Table S2 **Genetic diversity for 81 Cattle breeds.** Number of individuals per breed (N), mean number of alleles/locus (Am), mean effective number of alleles/locus (Ae), mean allelic richness per locus corrected for sample size (Ar), mean observed heterozygosity (Ho) and mean expected heterozygosity (He) and their standard deviations, within-breed inbreeding coefficient (FIS) and corresponding confidence interval.
(PDF)

Table S3 **Table S3. Estimated membership coefficients in each cluster (q), as inferred by STRUCTURE for K = 71.** Contribution of the more important cluster per breed is represented in bold.
(PDF)

Table S4 **Genetic contributions from Iberian, British, Continental European and Zebu breeds to Creole cattle.** Maximum-likelihood estimates of proportional genetic contributions from Iberian, British, Continental European and Zebu breeds to Creole cattle, considered as a whole or grouped in five different clusters. The SD was obtained from 1000 bootstrapping samples (over loci). **Creole cluster 1**: Guabalá, Guaymí, Romosinuano, Costeño con Cuernos; **Creole cluster 2**: Texas Longhorn, Criollo Baja California, Criollo Chihuahua, Criollo de Nayarit, Criollo Poblano, Sanmartinero; **Creole cluster 3**: Caracú, Criollo Argentino, Criollo Patagónico, Criollo Uruguayo; **Creole cluster 4:** Criollo Cubano, Siboney, Criollo de Ecuador, Velasquez, Caqueteño, Criollo de Chiapas, Criollo Pilcomayo, Criollo Casanareño, Chino Santandereano; **Creole cluster 5:** Pampa Chaqueño, Blanco Orejinegro, Lucerna, Hartón del Valle.
(DOC)

## References

[1] Stahl PW (2008) Animal Domestication in South America. In: Handbook of South American archaeology. Springer. 121–130.

[2] Crosby AW (1973) The Columbian Exchange: Biological and Cultural Consequences of 1492. Greenwood. 268 p.

[3] Rodero E, Rodero A, Delgado JV (1992) Primitive andalusian livestock an their implications in the discovery of America. Arch Zootec 41: 383–400.

[4] Rouse JE (1977) The Criollo: Spanish cattle in the Americas. University of Oklahoma Press. 303 p.

[5] Willham RL (1982) Genetic improvement of beef cattle in the United States: cattle, people and their interaction. J Anim Sci 54: 659–666. 7085522 10.2527/jas1982.543659x

[6] Santiago AA (1978) Evolution of Zebu cattle in Brazil. The Zebu Journal 1: 6.

[7] De Alba J (1987) Criollo Cattle of Latinamerica. In: Animal genetic resources. Strategies for improved use and conservation. Available:http://www.fao.org/docrep/010/ah806e/AH806E06.htm. Accessed 5 November 2011.

[8] Rischkowsky B, Pilling D, Commission on Genetic Resources for Food and Agriculture (2007) The state of the world’s animal genetic resources for food and agriculture. Roma: Food & Agriculture Organization of the United Nations (FAO). 554 p.

[9] Delgado JV, Martínez AM, Acosta A, Álvarez LA, Armstrong E, et al (2012) Genetic characterization of Latin-American Creole cattle using microsatellite markers. Anim Genet 43: 2–10 doi:10.1111/j.1365–2052.2011.02207.x 10.1111/j.1365-2052.2011.02207.x 22221019

[10] Cañón J, Alexandrino P, Bessa I, Carleos C, Carretero Y, et al (2001) Genetic diversity measures of local European beef cattle breeds for conservation purposes. Genet Sel Evol 33: 311–332 doi:10.1051/gse:2001121 11403750 10.1186/1297-9686-33-3-311 PMC2705410

[11] García D, Martínez A, Dunner S, Vega-Pla JL, Fernández C, et al (2006) Estimation of the genetic admixture composition of Iberian dry-cured ham samples using DNA multilocus genotypes. Meat Sci 72: 560–566 doi:10.1016/j.meatsci.2005.09.005 22061741 10.1016/j.meatsci.2005.09.005

[12] Tapio I, Värv S, Bennewitz J, Maleviciute J, Fimland E, et al (2006) Prioritization for conservation of northern European cattle breeds based on analysis of microsatellite data. Conserv Biol 20: 1768–1779 doi:10.1111/j.1523–1739.2006.00488.x 17181812 10.1111/j.1523-1739.2006.00488.x

[13] Ginja C, Telo Da Gama L, Penedo MCT (2010) Analysis of STR markers reveals high genetic structure in Portuguese native cattle. J Hered 101: 201–210 doi:10.1093/jhered/esp104 19965912 10.1093/jhered/esp104

[14] Li M-H, Kantanen J (2010) Genetic structure of Eurasian cattle (Bos taurus) based on microsatellites: clarification for their breed classification. Anim Genet 41: 150–158 doi:10.1111/j.1365–2052.2009.01980.x 10.1111/j.1365-2052.2009.01980.x 19845598

[15] Beja-Pereira A, Caramelli D, Lalueza-Fox C, Vernesi C, Ferrand N, et al (2006) The origin of European cattle: Evidence from modern and ancient DNA. Proc Natl Acad Sci USA 103: 8113–8118 doi:10.1073/pnas.0509210103 16690747 10.1073/pnas.0509210103 PMC1472438

[16] Edwards CJ, Ginja C, Kantanen J, Pérez-Pardal L, Tresset A, et al (2011) Dual Origins of Dairy Cattle Farming – Evidence from a Comprehensive Survey of European Y-Chromosomal Variation. PLoS ONE 6: e15922 doi:10.1371/journal.pone.0015922 21253012 10.1371/journal.pone.0015922 PMC3016991

[17] Gibbs RA, Taylor JF, Van Tassell CP, Barendse W, Eversole KA, et al (2009) Genome-wide survey of SNP variation uncovers the genetic structure of cattle breeds. Science 324: 528–532 doi:10.1126/science.1167936 19390050 10.1126/science.1167936 PMC2735092

[18] Gautier M, Laloë D, Moazami-Goudarzi K (2010) Insights into the genetic history of French cattle from dense SNP data on 47 worldwide breeds. PLoS ONE 5. Available:http://www.ncbi.nlm.nih.gov/pubmed/20927341. Accessed 11 November 2011. 10.1371/journal.pone.0013038 PMC2948016 20927341

[19] Lewis J, Abas Z, Dadousis C, Lykidis D, Paschou P, et al (2011) Tracing cattle breeds with principal components analysis ancestry informative SNPs. PLoS ONE 6: e18007 doi:10.1371/journal.pone.0018007 21490966 10.1371/journal.pone.0018007 PMC3072384

[20] Pritchard JK, Stephens M, Donnelly P (2000) Inference of population structure using multilocus genotype data. Genetics 155: 945–959. 10835412 10.1093/genetics/155.2.945 PMC1461096

[21] Wang J (2003) Maximum-likelihood estimation of admixture proportions from genetic data. Genetics 164: 747–765. 12807794 10.1093/genetics/164.2.747 PMC1462571

[22] Egito AA, Paiva SR, Albuquerque M do SM, Mariante AS, Almeida LD, et al (2007) Microsatellite based genetic diversity and relationships among ten Creole and commercial cattle breeds raised in Brazil. BMC Genet 8: 83 doi:10.1186/1471-2156-8-83 18067665 10.1186/1471-2156-8-83 PMC2228320

[23] Ulloa-Arvizu R, Gayosso-Vázquez A, Ramos-Kuri M, Estrada FJ, Montaño M, et al (2008) Genetic analysis of Mexican Criollo cattle populations. J Anim Breed Genet 125: 351–359 doi:10.1111/j.1439-0388.2008.00735.x 18803791 10.1111/j.1439-0388.2008.00735.x

[24] Martínez-Correal G, Alvarez LA, Martínez GC (2009) Conservación, Caracterización y Utilización de los bovinos Criollos en Colombia. In: X Simposio Iberoamericano sobre Conservación y Utilización de Recursos Zoogenéticos. Palmira, Colombia.

[25] Villalobos Cortés A, Martinez AM, Vega-Pla JL, Delgado JV (2009) Genetic characterization of the Guabala bovine population with microsatellites. Arch Zootec 58: 485–488.

[26] Villalobos Cortés AI, Martínez AM, Escobar C, Vega-Pla JL, Delgado JV (2010) Study of genetic diversity of the Guaymi and Guabala bovine populations by means of microsatellites. Livest Sci 131: 45–51 doi:10.1016/j.livsci.2010.02.024

[27] Martín-Burriel I, García-Muro E, Zaragoza P (1999) Genetic diversity analysis of six Spanish native cattle breeds using microsatellites. Anim Genet 30: 177–182. 10442978 10.1046/j.1365-2052.1999.00437.x

[28] Mateus JC, Penedo MCT, Alves VC, Ramos M, Rangel-Figueiredo T (2004) Genetic diversity and differentiation in Portuguese cattle breeds using microsatellites. Anim Genet 35: 106–113 doi:10.1111/j.1365–2052.2004.01089.x 15025569 10.1111/j.1365-2052.2004.01089.x

[29] Martín-Burriel I, Rodellar C, Lenstra JA, Sanz A, Cons C, et al (2007) Genetic diversity and relationships of endangered Spanish cattle breeds. J Hered 98: 687–691 doi:10.1093/jhered/esm096 17986470 10.1093/jhered/esm096

[30] Martín-Burriel I, Rodellar C, Cañón J, Cortés O, Dunner S, et al (2011) Genetic diversity, structure, and breed relationships in Iberian cattle. J Anim Sci 89: 893–906 doi:10.2527/jas.2010–3338 21415418 10.2527/jas.2010-3338

[31] Lara MAC, Contel EPB, Sereno JRB (2005) Genetic characterization of Zebu populations using molecular markers. Arch Zootec 206/207: 295–303.

[32] Dani MAC, Heinneman MB, Dani SU (2008) Brazilian Nelore cattle: a melting pot unfolded by molecular genetics. Genet Mol Res 7: 1127–1137. 19048491 10.4238/vol7-4gmr499

[33] European Cattle Genetic Diversity Consortium (2006) Marker-assisted conservation of European cattle breeds: An evaluation. Anim Genet 37: 475–481 doi:10.1111/j.1365–2052.2006.01511.x 16978177 10.1111/j.1365-2052.2006.01511.x

[34] Giovambattista G, Ripoli MV, De Luca JC, Mirol PM, Lirón JP, et al (2000) Male-mediated introgression of Bos indicus genes into Argentine and Bolivian Creole cattle breeds. Anim Genet 31: 302–305. 11105209 10.1046/j.1365-2052.2000.00658.x

[35] Ginja C, Penedo MCT, Melucci L, Quiroz J, Martínez López OR, et al (2010) Origins and genetic diversity of New World Creole cattle: inferences from mitochondrial and Y chromosome polymorphisms. Anim Genet 41: 128–141 doi:10.1111/j.1365–2052.2009.01976.x 19817725 10.1111/j.1365-2052.2009.01976.x

[36] Magee DA, Meghen C, Harrison S, Troy CS, Cymbron T, et al (2002) A partial african ancestry for the creole cattle populations of the Caribbean. J Hered 93: 429–432. 12642643 10.1093/jhered/93.6.429

[37] Carvajal-Carmona LG, Bermudez N, Olivera-Angel M, Estrada L, Ossa J, et al (2003) Abundant mtDNA diversity and ancestral admixture in Colombian criollo cattle (Bos taurus). Genetics 165: 1457–1463. 14668394 10.1093/genetics/165.3.1457 PMC1462844

[38] Lirón JP, Bravi CM, Mirol PM, Peral-García P, Giovambattista G (2006) African matrilineages in American Creole cattle: evidence of two independent continental sources. Anim Genet 37: 379–382 doi:10.1111/j.1365–2052.2006.01452.x 16879351 10.1111/j.1365-2052.2006.01452.x

[39] Achilli A, Bonfiglio S, Olivieri A, Malusà A, Pala M, et al (2009) The Multifaceted Origin of Taurine Cattle Reflected by the Mitochondrial Genome. PLoS ONE 4: e5753 doi:10.1371/journal.pone.0005753 19484124 10.1371/journal.pone.0005753 PMC2684589

[40] Uffo O, Martín-Burriel I, Martinez S, Ronda R, Osta R, et al (2006) Caracterización genética de seis proteínas lácteas en tras razas bovinas cubanas. AGRI 39: 15–24.

[41] Martinez AM, Llorente RV, Quiroz J, Martínez RD, Amstrong E, et al.. (2007) Estudio de la influencia de la raza bovina Marismeña en la formación de los bovinos Criollos. VIII Simposio Iberoamericano sobre Conservación y utilización de Recursos Zoogenéticos. Quevedo, Ecuador.

[42] Miretti MM, Dunner S, Naves M, Contel EP, Ferro JA (2004) Predominant African-derived mtDNA in Caribbean and Brazilian Creole cattle is also found in Spanish cattle (Bos taurus). J Hered 95: 450–453 doi:10.1093/jhered/esh070 15388773 10.1093/jhered/esh070

[43] Gautier M, Naves M (2011) Footprints of selection in the ancestral admixture of a New World Creole cattle breed. Molecular Ecology 20: 3128–3143 doi:10.1111/j.1365-294X.2011.05163.x 21689193 10.1111/j.1365-294X.2011.05163.x

[44] MacHugh DE, Shriver MD, Loftus RT, Cunningham P, Bradley DG (1997) Microsatellite DNA Variation and the Evolution, Domestication and Phylogeography of Taurine and Zebu Cattle (Bos Taurus and Bos Indicus). Genetics 146: 1071–1086. 9215909 10.1093/genetics/146.3.1071 PMC1208036

[45] Mirol PM, Giovambattista G, Lir|[oacute]|n JP, Dulout FN (2003) African and European mitochondrial haplotypes in South American Creole cattle. Heredity 91: 248–254 doi:10.1038/sj.hdy.6800312 12939625 10.1038/sj.hdy.6800312

[46] Lush JL (1943) Animal Breeding Plans. Ames, Iowa, USA: The Iowa State College Press. 457 p.

[47] Villalobos Cortés A, Martinez AM, Vega-Pla JL, Delgado JV (2009) History of Panama bovines and their relationships with other Iberoamerican popultions. Arch Zootec 58: 121–129.

[48] Primo AT (2004) América: conquista e colonização: a fantástica história dos conquistadores ibéricos e seus animais na era dos descobrimentos. Porto Alegre, Brazil: Movimento. 192 p.

[49] Martínez AM, Calderón J, Camacho E, Rico C, Vega-Pla JL, et al (2005) Genetic characterisation of the Mostrenca cattle with microsatellites. Arch Zootec 206: 357–361.

[50] FAO (2004) Secondary Guidelines for Development of National Farm Animal Genetic Resources Management Plans: Management of Small Populations at Risk. Roma: Food & Agriculture Organization of the United Nations (FAO). 225 p.

[51] Martínez AM, Delgado JV, Rodero A, Vega-Pla JL (2000) Genetic structure of the Iberian pig breed using microsatellites. Anim Genet 31: 295–301. 11105208 10.1046/j.1365-2052.2000.00645.x

[52] Nei M (1973) Analysis of gene diversity in subdivided populations. Proc Natl Acad Sci USA 70: 3321–3323. 4519626 10.1073/pnas.70.12.3321 PMC427228

[53] Park SDE (2001) The Excel Microsatellite Toolkit. Trypanotolerance in West African Cattle and the Population Genetic Effects of Selection [Ph.D. thesis ]. University of Dublin. Available:http://animalgenomics.ucd.ie/sdepark/ms-toolkit/. Accessed 5 November 2011.

[54] Weir BS, Cockerham CC (1984) Estimating F-Statistics for the Analysis of Population Structure. Evolution 38: 1358–1370 doi:10.2307/2408641 28563791 10.1111/j.1558-5646.1984.tb05657.x

[55] Belkhir K, Borsa P, Chikhi L, Raufaste N, Bonhomme F (2004) GENETIX 4.05, logiciel sous Windows TM pour la génétique des populations. Laboratoire Génome, Populations, Interactions, CNRS UMR 5000, Université de Montpellier II, Montpellier (France). GENETIX INTRODUCTION. Available:http://www.genetix.univ-montp2.fr/genetix/intro.htm. Accessed 5 November 2011.

[56] Yeh FC, Boyle TJB (1997) Population genetic analysis of co-dominant and dominant markers and quantitative traits. Belg J Bot 129: 157.

[57] Goudet J (1995) FSTAT, a program to estimate and test gene diversities and fixation indices. Department of Ecology & Evolution, Biology Building, UNIL, CH-1015 LAUSANNE, Switzerland. Available:http://www2.unil.ch/popgen/softwares/fstat.htm. Accessed 5 November 2011.

[58] Raymond M, Rousset F (1995) GENEPOP (Version 1.2): Population Genetics Software for Exact Tests and Ecumenicism. J Hered 86: 248–249.

[59] Guo SW, Thompson EA (1992) Performing the exact test of Hardy-Weinberg proportion for multiple alleles. Biometrics 48: 361–372. 1637966

[60] Excoffier L, Smouse PE, Quattro JM (1992) Analysis of molecular variance inferred from metric distances among DNA haplotypes: application to human mitochondrial DNA restriction data. Genetics 131: 479–491. 1644282 10.1093/genetics/131.2.479 PMC1205020

[61] Excoffier L, Laval G, Schneider S (2005) Arlequin (version 3.0): an integrated software package for population genetics data analysis. Evol Bioinform Online 1: 47–50. PMC2658868 19325852

[62] Reynolds J, Weir BS, Cockerham CC (1983) Estimation of the coancestry coefficient: basis for a short-term genetic distance. Genetics 105: 767–779. 17246175 10.1093/genetics/105.3.767 PMC1202185

[63] Langella O (1999) Populations 1.2.31 CNRS UPR9034. Available:http://www.bioinformatics.org/~tryphon/populations/. Accessed 5 November 2011.

[64] Huson DH, Bryant D (2006) Application of phylogenetic networks in evolutionary studies. Mol Biol Evol 23: 254–267 doi:10.1093/molbev/msj030 16221896 10.1093/molbev/msj030

[65] Lebart L, Morineau A, Warwick KM (1984) Multivariate descriptive statistical analysis: correspondence analysis and related techniques for large matrices. Wiley. 266 p.

[66] Evanno G, Regnaut S, Goudet J (2005) Detecting the number of clusters of individuals using the software STRUCTURE: a simulation study. Mol Ecol 14: 2611–2620 doi:10.1111/j.1365-294X.2005.02553.x 15969739 10.1111/j.1365-294X.2005.02553.x

[67] Rosenberg NA (2004) DISTRUCT: a program for the graphical display of population structure. Molecular Ecology Notes 4: 137–138 doi:10.1046/j.1471–8286.2003.00566.x

