# Supplementary figures and images for: Genetic Footprints of Iberian Cattle in America 500 Years after the Arrival of Columbus
Source: PLoS One. 2012 Nov 14;7(11):e49066. doi: 10.1371/journal.pone.0049066 (PMC3498335; doi:10.1371/journal.pone.0049066)

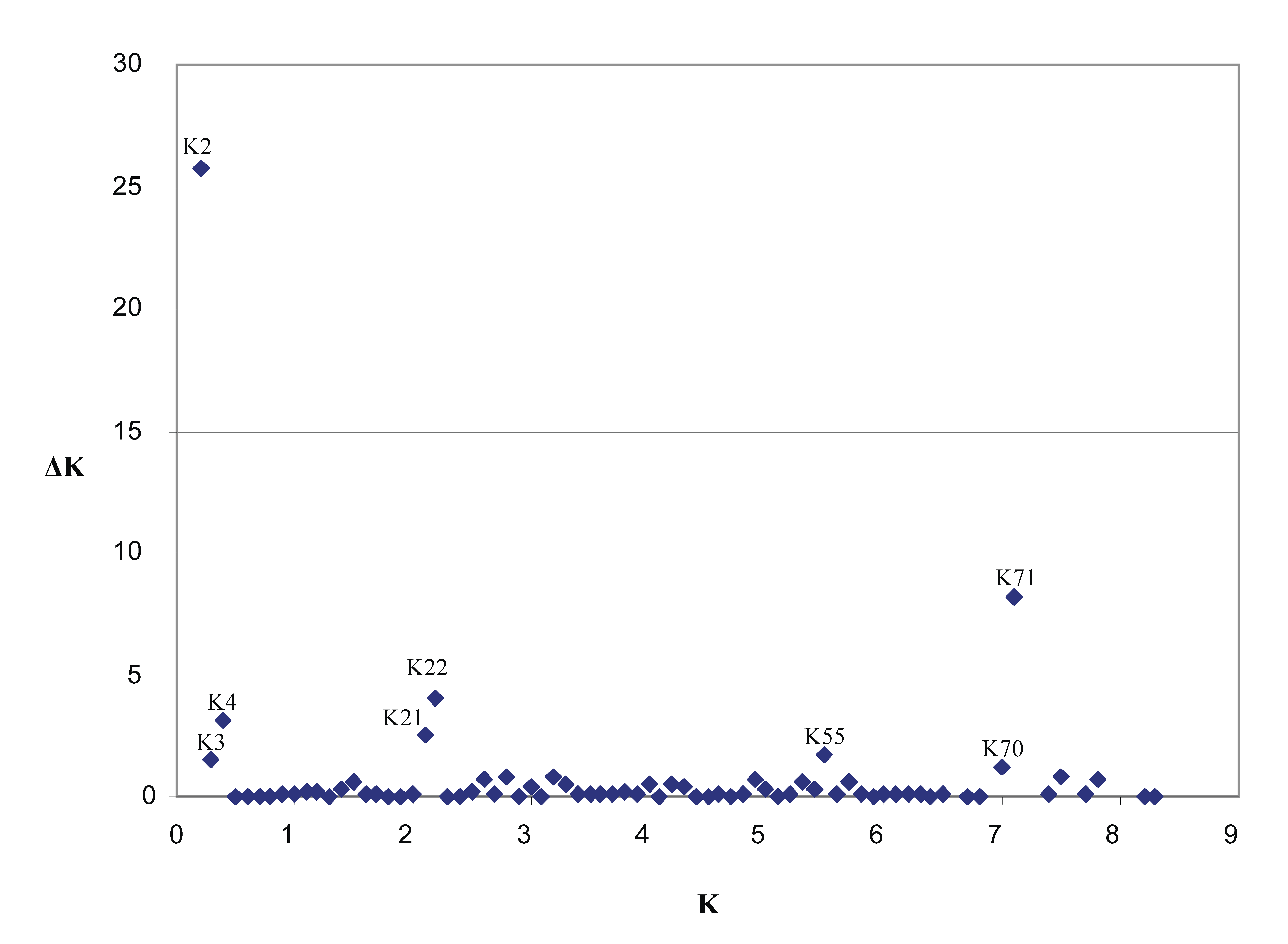

Supplement: Figure S1 — Graphical representation of ΔK values for K = 2 to K = 81. Representation for 81 Cattle breeds based on STRUCTURE results following Evanno criterion. (TIF) [file pone.0049066.s001.tif]

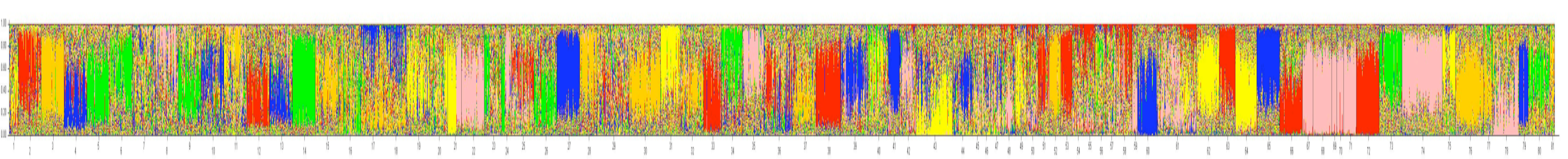

Supplement: Figure S2 — Population structure of 81 cattle breeds using STRUCTURE when K = 71. Graphical representation of individual genotype membership coefficients (q) when K = 71. Each animal is represented by a single vertical line divided into 71 coloured segments using only 6 colours showing the individual’s estimated membership proportions in that cluster. 1: Betizu (BET), 2: Toro de Lidia (TL), 3: Menorquina (MEN), 4: Alistana (ALS), 5: Sayaguesa (SAY), 6: Tudanca (TUD), 7: Asturiana de los Valles (ASV), 8: Asturiana de las Montañas (ASM), 9: Retinta (RET), 10: Morucha (MOR), 11: Avileña (AVI), 12: Pirenaica (PIRM), 13: Rubia Gallega (RGA), 14: Mallorquina (MALL), 15: Monchina (MON), 16: Serrana de Teruel (STE), 17: Parda de Montaña (PM), 18: Bruna de los Pirineos (BRP), 19: Pasiega (PAS), 20: Berrenda en Colorado (BC), 21: Berrenda en Negro (BN), 22: Marismeña (MAR), 23: Pajuna (PAJ), 24: Negra Andaluza (NAN), 25: Vaca Canaria (VCA), 26: Vaca Palmera (PAL), 27: Alentejana (ALT), 28: Arouquesa (ARO), 29: Barrosã (BARR), 30: Brava de Lide (BRAV), 31: Cachena (CACH), 32: Garvonesa (GARV), 33: Marinhoa (MARI), 34: Maronesa (MARO), 35: Mertolenga (MERT), 36: Minhota (MINH), 37: Mirandesa (MIRA), 38: Preta (PRET), 39: Ramo Grande (RG); CREOLE. 40: Guabalá (GUA), 41: Guaymí (GY), 42: Texas Longhorn (TLH), 43: Criollo Poblano (CPO), 44: Criollo de Baja California (CBC), 45: Criollo de Chihuahua (CHU), 46: Criollo de Nayarit (CNY), 47: Criollo de Chiapas (CHI), 48: Blanco Orejinegro (BON), 49: Caqueteño (CAQ), 50: Sanmartinero (SM), 51: Romosinuano (RMS), 52: Costeño con Cuernos (CCC), 53: Chino Santandereano (CH), 54: Velasquez (VEL), 55: Lucerna (LUC), 56: Hartón del Valle (HV), 57: Criollo Casanareño (CC), 58: Criollo Ecuatoriano (EC), 59: Criollo Uruguayo (CUR), 60: Pampa Chaqueño (PA), 61: Criollo Pilcomayo (PIL), 62: Criollo Argentino (CARG), 63: Criollo Patagónico (PAT), 64: Caracú (CAR), 65: Cubano (CUB), 66: Siboney (SIB); ZEBU: 67: Gyr (GYR), 68: Brahman (BRH), 69: Sindi (SIN), 7 [file pone.0049066.s002.tif]
