# Supplementary material for: Genetic Footprints of Iberian Cattle in America 500 Years after the Arrival of Columbus
Source: PLoS One. 2012 Nov 14;7(11):e49066. doi: 10.1371/journal.pone.0049066 (PMC3498335; doi:10.1371/journal.pone.0049066)
Supplement: Table S1 — Breeds, samples and origins. Breed names, acronyms (Acron.), sample sizes (N), sample type, breed type, genetic group (GG), country of sampling and region of origin (Reg) of the 81 breeds included in this study. (PDF) [file pone.0049066.s003.pdf]

| Breed name | Acron. | N | Sample type | Breed type | GG | Sample origin | Reg |
|---|---|---|---|---|---|---|---|
| Criollo Argentino | CARG | 50 | Hair roots | Creole | Taurine | Argentina | SA |
| Criollo Patagónico | PAT | 35 | Hair roots | Creole | Taurine | Argentina | SA |
| Caracú | CAR | 47 | Hair roots | Creole | Taurine | Brasil | SA |
| Blanco Orejinegro | BON | 25 | Blood | Creole | Taurine | Colombia | SA |
| Caqueteño | CAQ | 25 | Blood | Creole | Taurine | Colombia | SA |
| Criollo Casanareño | CC | 35 | Blood | Creole | Taurine | Colombia | SA |
| Chino Santandereano | CH | 25 | Blood | Creole | Taurine | Colombia | SA |
| Costeño con Cuernos | CCC | 25 | Blood | Creole | Taurine | Colombia | SA |
| Hartón del Valle | HV | 22 | Blood | Creole | Taurine | Colombia | SA |
| Lucerna | LUC | 24 | Blood | Creole | Taurine | Colombia | SA |
| Romosinuano | RMS | 25 | Blood | Creole | Taurine | Colombia | SA |
| Sanmartinero | SM | 25 | Blood | Creole | Taurine | Colombia | SA |
| Velasquez | VEL | 25 | Blood | Creole | Taurine | Colombia | SA |
| Cubano | CUB | 50 | Blood | Creole | Taurine | Cuba | CI |
| Siboney | SIB | 50 | Blood | Creole | Taurine | Cuba | CI |
| Criollo Ecuatoriano | EC | 12 | Hair roots | Creole | Taurine | Ecuador | SA |
| Criollo de Baja California | CBC | 21 | Hair roots | Creole | Taurine | México | NA |
| Criollo de Chiapas | CHI | 30 | Hair roots | Creole | Taurine | México | NA |
| Criollo de Chihuahua | CHU | 19 | Hair roots | Creole | Taurine | México | NA |
| Criollo de Nayarit | CNY | 24 | Hair roots | Creole | Taurine | México | NA |
| Criollo Poblano | CPO | 43 | Hair roots | Creole | Taurine | México | NA |
| Guabalá | GUA | 25 | Hair roots | Creole | Taurine | Panama | CA |
| Guaymí | GY | 36 | Hair roots | Creole | Taurine | Panama | CA |
| Pampa Chaqueño | PA | 50 | Hair roots | Creole | Taurine | Paraguay | SA |
| Criollo Pilcomayo | PIL | 36 | Hair roots | Creole | Taurine | Paraguay | SA |
| Criollo Uruguayo | CUR | 43 | Blood | Creole | Taurine | Uruguay | SA |
| Texas Longhorn | TLH | 80 | Hair roots, Semen | Creole | Taurine | USA | NA |

| | | | | | | | |
|---|---|---|---|---|---|---|---|
| Alistana | ALS | 50 | Blood | Native | Taurine | Spain | IB |
| Asturiana de las Montañas | ASM | 50 | Blood | Native | Taurine | Spain | IB |
| Asturiana de los Valles | ASV | 50 | Blood | Native | Taurine | Spain | IB |
| Avileña | AVI | 50 | Blood | Native | Taurine | Spain | IB |
| Berrenda en Colorado | BC | 40 | Blood | Native | Taurine | Spain | IB |
| Berrenda en Negro | BN | 30 | Blood | Native | Taurine | Spain | IB |
| Betizu | BET | 20 | Blood | Native | Taurine | Spain | IB |
| Bruna de los Pirineos | BRP | 50 | Blood | Native | Taurine | Spain | IB |
| Mallorquina | MALL | 50 | Blood | Native | Taurine | Spain, Balearic Islands | IB |
| Menorquina | MEN | 50 | Blood | Native | Taurine | Spain, Balearic Islands | IB |
| Monchina | MON | 50 | Blood | Native | Taurine | Spain | IB |
| Morucha | MOR | 50 | Blood | Native | Taurine | Spain | IB |
| Marismeña | MAR | 50 | Blood | Native | Taurine | Spain | IB |
| Negra Andaluza | NAN | 21 | Hair roots | Native | Taurine | Spain | IB |
| Pajuna | PAJ | 38 | Blood | Native | Taurine | Spain | IB |
| Parda de Montaña | PM | 50 | Blood | Native | Taurine | Spain | IB |
| Pasiega | PAS | 50 | Blood | Native | Taurine | Spain | IB |
| Pirenaica | PIRM | 50 | Blood | Native | Taurine | Spain | IB |
| Retinta | RET | 50 | Hair roots | Native | Taurine | Spain | IB |
| Rubia Gallega | RGA | 50 | Hair roots | Native | Taurine | Spain | IB |
| Sayaguesa | SAY | 50 | Blood | Native | Taurine | Spain | IB |
| Serrana de Teruel | STE | 50 | Blood | Native | Taurine | Spain | IB |
| Toro de Lidia | TL | 50 | Blood | Native | Taurine | Spain | IB |
| Tudanca | TUD | 50 | Blood | Native | Taurine | Spain | IB |
| Vaca Canaria | VCA | 50 | Blood | Native | Taurine | Spain, Canary Islands | IB |
| Vaca Palmera | PAL | 50 | Blood | Native | Taurine | Spain, Canary Islands | IB |
| Alentejana | ALT | 38 | Blood | Native | Taurine | Portugal | IB |
| Arouquesa | ARO | 70 | Blood | Native | Taurine | Portugal | IB |

| | | | | | | | |
|---|---|---|---|---|---|---|---|
| Barrosã | BARR | 69 | Blood | Native | Taurine | Portugal | IB |
| Brava de Lide | BRAV | 43 | Blood | Native | Taurine | Portugal | IB |
| Cachena | CACH | 51 | Blood | Native | Taurine | Portugal | IB |
| Garvonesa | GARV | 39 | Blood | Native | Taurine | Portugal | IB |
| Marinhoa | MARI | 46 | Blood | Native | Taurine | Portugal | IB |
| Maronesa | MARO | 47 | Blood | Native | Taurine | Portugal | IB |
| Mertolenga | MERT | 64 | Blood | Native | Taurine | Portugal | IB |
| Minhota | MINH | 50 | Blood | Native | Taurine | Portugal | IB |
| Mirandesa | MIRA | 54 | Blood | Native | Taurine | Portugal | IB |
| Preta | PRET | 60 | Blood | Native | Taurine | Portugal | IB |
| Ramo Grande | RG | 44 | Blood | Native | Taurine | Portugal, Azores Islands | IB |
| Aberdeen Angus | AA | 62 | Hair roots, Semen | Commercial | Taurine | Argentina; USA | BI |
| British White | BWC | 19 | Hair roots, Semen | Native | Taurine | USA | BI |
| Hereford | HER | 88 | Hair roots, Semen | Commercial | Taurine | Argentina, México, USA | BI |
| Jersey | JER | 20 | Hair roots, Semen | Commercial | Taurine | USA | BI |
| Shorthorn | SH | 11 | Hair roots, Semen | Commercial | Taurine | USA | BI |
| Charolais | CHAR | 58 | Blood | Commercial | Taurine | Portugal | CE |
| Friesian | FRI | 50 | Blood | Commercial | Taurine | Portugal | CE |
| Limousin | LIM | 47 | Hair roots | Commercial | Taurine | Portugal | CE |
| Brown Swiss | BSW | 29 | Hair roots | Commercial | Taurine | México | CE |
| Brahman | BRH | 41 | Hair roots, Semen | Commercial | Indicine | México, USA | IN |
| Gyr | GYR | 23 | Blood | Commercial | Indicine | Brazil | IN |
| Guzerat | GUZ | 15 | Hair roots | Commercial | Indicine | Brazil | IN |
| Nelore | NEL | 28 | Hair roots | Commercial | Indicine | Brazil | IN |
| Sindi | SIN | 11 | Hair roots | Commercial | Indicine | Brazil | IN |
| Zebu Cubano | CUZ | 50 | Blood | Commercial | Indicine | Cuba | IN |
