# Supplementary material for: Genetic Footprints of Iberian Cattle in America 500 Years after the Arrival of Columbus
Source: PLoS One. 2012 Nov 14;7(11):e49066. doi: 10.1371/journal.pone.0049066 (PMC3498335; doi:10.1371/journal.pone.0049066)
Supplement: Table S2 — Genetic diversity for 81 Cattle breeds. Number of individuals per breed (N), mean number of alleles/locus (Am), mean effective number of alleles/locus (Ae), mean allelic richness per locus corrected for sample size (Ar), mean observed heterozygosity (Ho) and mean expected heterozygosity (He) and their standard deviations, within-breed inbreeding coefficient (FIS) and corresponding confidence interval. (PDF) [file pone.0049066.s004.pdf]

BREED	N	Am (SD)	Ae	Ar	Ho (SD)	He (SD)	Fis (CI)
**Creole**							
CARG	50	6.26 (1.66)	3.326	4.003	0.673 (0.015)	0.678 (0.023)	0.007 (-0.049/0.041)
PAT	35	5.32 (1.57)	3.224	3.841	0.629 (0.019)	0.670 (0.025)	0.062 (-0.012/0.104)
CAR	47	6.74 (1.73)	3.695	4.321	0.733 (0.015)	0.711 (0.022)	-0.030 (-0.080/-0.006)
BON	25	5.74 (1.76)	3.391	4.100	0.737 (0.020)	0.697 (0.023)	-0.059 (-0.138/-0.027)
CAQ	25	7.58 (1.57)	4.778	5.220	0.780 (0.019)	0.787 (0.017)	0.009 (-0.070/0.040)
SM	25	6.37 (1.16)	3.597	4.391	0.692 (0.022)	0.721 (0.018)	0.042 (-0.046/0.076)
RMS	25	5.11 (1.59)	3.212	3.941	0.651 (0.023)	0.669 (0.030)	0.028 (-0.066/0.071)
CCC	25	5.26 (1.37)	3.288	3.941	0.692 (0.022)	0.671 (0.031)	-0.032 (-0.120/0.007)
CH	25	7.32 (1.73)	4.377	5.028	0.726 (0.021)	0.776 (0.013)	0.066 (-0.004/0.089)
VEL	25	6.79 (1.44)	4.307	4.918	0.730 (0.021)	0.769 (0.016)	0.053 (-0.033/0.086)
LUC	24	6.63 (2.06)	3.820	4.688	0.673 (0.024)	0.717 (0.025)	0.063 (-0.027/0.092)
HV	22	7.74 (1.73)	4.600	5.238	0.783 (0.021)	0.783 (0.016)	-0.001(-0.0867/0.035)
CC	35	8.00 (1.65)	3.524	4.125	0.739 (0.019)	0.766 (0.020)	0.035 (-0.022/0.060)
CUB	50	7.58 (2.36)	4.614	4.920	0.793 (0.013)	0.761 (0.018)	-0.043 (-0.084/-0.024)
SIB	50	8.05 (2.30)	4.728	5.051	0.746 (0.014)	0.762 (0.027)	0.022 (-0.027/0.049)
EC	12	6.63 (2.11)	4.299	5.233	0.732 (0.029)	0.771 (0.023)	0.054 (-0.074/0.076)
CPO	43	8.37 (2.01)	4.672	5.075	0.693 (0.016)	0.774 (0.017)	0.105 (0.052/0.133)
CBC	21	7.05 (1.58)	4.183	4.983	0.742 (0.024)	0.760 (0.019)	0.025 (-0.071/0.048)
CHU	19	6.68 (1.49)	4.333	5.140	0.719 (0.026)	0.777 (0.018)	0.077 (-0.027/0.105)
CNY	24	7.74 (1.94)	4.718	5.248	0.749 (0.021)	0.788 (0.018)	0.050 (-0.019/0.066)
CHI	30	7.84 (1.57)	4.768	5.234	0.741 (0.019)	0.782 (0.021)	0.053 (-0.013/0.082)
GUA	25	5.79 (1.96)	3.531	4.104	0.629 (0.022)	0.660 (0.045)	0.048 (-0.047/0.093)
GY	36	7.79 (1.65)	4.263	4.925	0.735 (0.017)	0.756 (0.017)	0.028 (-0.031/0.053)
PA	50	8.11 (1.79)	4.624	5.050	0.750 (0.014)	0.771 (0.017)	0.028 (-0.015/0.051)
PIL	36	7.53 (1.74)	4.682	5.071	0.764 (0.016)	0.768 (0.022)	0.006 (-0.043/0.023)
CUR	43	5.63 (1.67)	3.222	3.974	0.668 (0.017)	0.674 (0.019)	0.009 (-0.046/0.035)
TLH	80	8.05 (2.46)	4.390	4.784	0.707 (0.012)	0.740 (0.025)	0.045 (0.010/0.067)
**Spanish**							
ALS	50	7.37 (1.67)	3.777	4.406	0.664 (0.015)	0.711 (0.025)	0.068 (0.012/0.102)
ASM	50	7.16 (2.22)	3.985	4.569	0.674 (0.015)	0.723 (0.025)	0.069 (0.018/0.101)
ASV	50	8.63 (2.17)	4.618	5.069	0.728 (0.014)	0.754 (0.024)	0.034 (-0.012/0.058)
AVI	50	7.37 (2.43)	4.220	4.701	0.688 (0.015)	0.731 (0.028)	0.060 (0.014/0.087)

BC	40	7.68 (2.16)	4.731	5.109	0.731 (0.016)	0.779 (0.016)	0.063 (0.005/0.098)
BN	30	5.21 (1.55)	2.966	3.800	0.595 (0.021)	0.635 (0.028)	0.064 (-0.022/0.105)
BET	20	5.68 (1.45)	3.703	4.424	0.665 (0.026)	0.730 (0.019)	0.092 (-0.041/0.159)
BRP	50	7.53 (2.09)	3.782	4.458	0.687 (0.015)	0.697 (0.030)	0.014 (-0.033/0.040)
MALL	50	4.37 (1.54)	2.645	3.171	0.542 (0.016)	0.577 (0.045)	0.061 (0.001/0.099)
MAR	50	5.42 (1.39)	2.703	3.573	0.602 (0.016)	0.608 (0.027)	0.010 (-0.042/0.040)
MEN	50	5.84 (1.98)	3.005	3.782	0.636 (0.016)	0.628 (0.031)	-0.013 (-0.071/0.024)
MON	50	8.11 (2.28)	4.330	4.882	0.732 (0.014)	0.751 (0.020)	0.026 (-0.025/0.057)
MOR	50	7.74 (1.76)	4.293	4.810	0.704 (0.015)	0.754 (0.020)	0.067 (0.025/0.088)
NA	21	6.16 (2.14)	3.685	4.454	0.634 (0.025)	0.708 (0.027)	0.107 (-0.003/0.146)
PAJ	38	7.37 (2.17)	4.156	4.691	0.711 (0.017)	0.741 (0.022)	0.042 (-0.015/0.073)
PM	50	7.47 (1.81)	3.679	4.488	0.700 (0.015)	0.710 (0.020)	0.014 (-0.032/0.038)
PAS	50	7.53 (2.20)	4.024	4.669	0.686 (0.015)	0.721 (0.026)	0.049 (0.002/0.075)
PIRM	50	7.16 (2.06)	3.883	4.566	0.704 (0.015)	0.725 (0.019)	0.029 (-0.017/0.055)
RET	50	7.53 (2.61)	4.472	4.834	0.736 (0.014)	0.758 (0.022)	0.031 (-0.019/0.059)
RGA	50	7.47 (1.95)	3.977	4.566	0.696 (0.015)	0.713 (0.032)	0.024 (-0.017/0.043)
SAY	50	7.32 (1.60)	4.177	4.733	0.686 (0.015)	0.743 (0.022)	0.078 (0.025/0.111)
STE	50	7.95 (1.99)	4.559	4.943	0.727 (0.014)	0.762 (0.021)	0.045 (-0.001/0.071)
TL	50	6.32 (2.11)	3.286	4.029	0.587 (0.017)	0.651 (0.034)	0.099 (0.040/0.135)
TUD	50	7.16 (2.34)	3.947	4.502	0.659 (0.015)	0.696 (0.030)	0.054 (0.011/0.078)
VCA	50	8.00 (2.29)	4.463	4.923	0.729 (0.015)	0.768 (0.016)	0.052 (0.005/0.076)
PAL	50	5.58 (1.84)	3.111	3.757	0.633 (0.016)	0.626 (0.040)	-0.011 (-0.058/0.017)
**Portuguese**							
ALT	38	5.79 (1.32)	3.377	4.084	0.648 (0.018)	0.688 (0.024)	0.059 (0.001/0.091)
ARO	70	8.05 (2.61)	4.328	4.779	0.717 (0.012)	0.745 (0.024)	0.038 (-0.004/0.065)
BARR	69	6.53 (1.71)	3.691	4.265	0.697 (0.013)	0.696 (0.034)	-0.001 (-0.043/0.028)
BRAV	43	5.47 (1.81)	3.098	3.709	0.565 (0.017)	0.631 (0.037)	0.107 (0.042/0.148)
CACH	51	7.68 (1.95)	4.172	4.689	0.728 (0.014)	0.727 (0.034)	-0.001 (-0.043/0.021)
GARV	39	6.16 (1.89)	3.234	3.968	0.677 (0.017)	0.651 (0.034)	-0.041 (-0.096/-0.014)
MARI	46	6.11 (1.73)	3.548	4.153	0.689 (0.016)	0.692 (0.029)	0.005 (-0.046/0.034)
MARO	47	6.68 (1.63)	3.548	4.276	0.685 (0.016)	0.693 (0.027)	0.012 (-0.037/0.040)
MERT	64	7.74 (2.45)	4.006	4.593	0.641 (0.014)	0.722 (0.028)	0.113 (0.066/0.145)
MINH	50	7.68 (2.24)	3.898	4.635	0.767 (0.014)	0.725 (0.024)	-0.059 (-0.099/-0.039)
MIRA	54	5.63 (1.07)	2.813	3.546	0.615 (0.015)	0.623 (0.026)	0.012 (-0.041/0.046)

PRET	60	7.05 (2.61)	3.550	4.256	0.653 (0.014)	0.674 (0.036)	0.032 (-0.017/0.065)
RG	44	7.53 (2.39)	3.852	4.567	0.688 (0.016)	0.721 (0.023)	0.045 (-0.014/0.086)
**Continental European**							
FRI	50	6.89 (2.40)	3.920	4.512	0.732 (0.015)	0.714 (0.025)	-0.025 (-0.075/0.005)
SPA	29	6.79 (2.15)	3.860	4.425	0.739 (0.019)	0.726 (0.024)	-0.018 (-0.088/0.011)
CHAR	58	6.95 (1.99)	3.904	3.896	0.682 (0.014)	0.705 (0.032)	0.033 (-0.012/0.062)
LIM	47	6.95 (1.99)	4.111	3.696	0.740 (0.015)	0.743 (0.018)	0.004 (-0.041/0.029)
**British**							
AA	62	6.26 (1.88)	3.510	4.447	0.633 (0.014)	0.682 (0.034)	0.072 (0.025/0.101)
BWC	19	5.00 (1.29)	3.242	4.137	0.667 (0.026)	0.676 (0.028)	0.013 (-0.139/0.106)
HER	88	6.63 (1.54)	3.556	4.735	0.655 (0.012)	0.704 (0.018)	0.070 (0.033/0.095)
JER	20	4.79 (1.03)	2.906	4.611	0.666 (0.024)	0.648 (0.026)	-0.029 (-0.124/0.011)
SH	11	5.16 (1.98)	3.067	4.120	0.690 (0.032)	0.656 (0.033)	-0.056 (-0.194/-0.035)
**Zebu**							
BRH	41	7.74 (2.40)	3.620	4.447	0.682 (0.017)	0.699 (0.024)	0.025 (-0.024/0.047)
GUZ	15	5.05 (1.61)	2.957	4.646	0.606 (0.029)	0.643 (0.028)	0.060 (-0.081/0.119)
GYR	23	5.79 (1.65)	3.143	4.008	0.607 (0.025)	0.658 (0.031)	0.079 (-0.022/0.114)
NEL	28	5.53 (1.58)	2.701	4.226	0.544 (0.022)	0.603 (0.028)	0.100 (0.018/0.144)
SIN	11	5.42 (1.77)	3.179	3.627	0.651 (0.034)	0.672 (0.030)	0.033 (-0.117/0.039)
CUZ	50	7.53 (2.06)	4.279	5.514	0.713 (0.015)	0.710 (0.041)	-0.003 (-0.046/0.019)
Overall							
Mean	41.15	6.78 (1.88)	3.820	4.482	0.688 (0.018)	0.711 (0.025)	0.033 (-0-033/0.063)
