# Supplementary material for: Genetic Footprints of Iberian Cattle in America 500 Years after the Arrival of Columbus
Source: PLoS One. 2012 Nov 14;7(11):e49066. doi: 10.1371/journal.pone.0049066 (PMC3498335; doi:10.1371/journal.pone.0049066)
Supplement: Table S4 — Genetic contributions from Iberian, British, Continental European and Zebu breeds to Creole cattle. Maximum-likelihood estimates of proportional genetic contributions from Iberian, British, Continental European and Zebu breeds to Creole cattle, considered as a whole or grouped in five different clusters. The SD was obtained from 1000 bootstrapping samples (over loci). Creole cluster 1: Guabalá, Guaymí, Romosinuano, Costeño con Cuernos; Creole cluster 2: Texas Longhorn, Criollo Baja California, Criollo Chihuahua, Criollo de Nayarit, Criollo Poblano, Sanmartinero; Creole cluster 3: Caracú, Criollo Argentino, Criollo Patagónico, Criollo Uruguayo; Creole cluster 4: Criollo Cubano, Siboney, Criollo de Ecuador, Velasquez, Caqueteño, Criollo de Chiapas, Criollo Pilcomayo, Criollo Casanareño, Chino Santandereano; Creole cluster 5: Pampa Chaqueño, Blanco Orejinegro, Lucerna, Hartón del Valle. (DOC) [file pone.0049066.s006.doc]

| **GROUP** | **All Creole breeds** | **Cluster 1** | **Cluster 2** | **Cluster 3** | **Cluster 4** | **Cluster 5** |
| --- | --- | --- | --- | --- | --- | --- |
| Iberian | 0.6194±0.0785 | 0.8775±0.1630 | 0.7072±0.1201 | 0.5813±0.1022 | 0,5562±0,1926 | 0.2864±0.0631 |
| British | 0.1034±0.0381 | -0.0979±0.0998 | 0.0184±0.0855 | 0.3662±0.0729 | -0.0616±0.2500 | 0,3772±0.1023 |
| Continental European | 0.1108±0.0706 | 0.1308±0.1379 | 0.1493±0.1014 | 0.0154±0.0820 | 0.1032±0.1225 | 0,2524±0.1321 |
| *Bos indicus* | 0.1664±0.0181 | 0.0897±0.0256 | 0.1250±0.0259 | 0.0839±0.0290 | 0.3406±0.0432 | 0,0839 ±0.0631 |
